# Global, Regional, and National Time Trends in Mortality for Ischemic Heart Disease, 1990–2019: An Age-Period-Cohort Analysis for the Global Burden of Disease 2019 Study

**DOI:** 10.31083/RCM45099

**Published:** 2025-09-30

**Authors:** Xuanqi An, Ning Zhou, Jing Xie, Chuanxu Liu, Fengwen Zhang, Wenbin Ouyang, Shouzheng Wang, Zeye Liu, Xiangbin Pan

**Affiliations:** ^1^Department of Structural Heart Disease, National Center for Cardiovascular Disease, China & Fuwai Hospital, Chinese Academy of Medical Sciences & Peking Union Medical College, 100037 Beijing, China; ^2^National Health Commission Key Laboratory of Cardiovascular Regeneration Medicine, 100037 Beijing, China; ^3^Key Laboratory of Innovative Cardiovascular Devices, Chinese Academy of Medical Sciences, 100037 Beijing, China; ^4^National Clinical Research Center for Cardiovascular Diseases, Fuwai Hospital, Chinese Academy of Medical Sciences, 100037 Beijing, China; ^5^State Key Laboratory of Cardiovascular Disease, Fuwai Hospital, National Center for Cardiovascular Diseases, Fuwai Hospital, Chinese Academy of Medical Sciences, and Peking Union Medical College, 100037 Beijing, China; ^6^Emergency Department, National Clinical Research Center of Cardiovascular Diseases, Fuwai Hospital, National Center for Cardiovascular Diseases, Chinese Academy of Medical Sciences and Peking Union Medical College, 100037 Beijing, China; ^7^Department of Pharmacy, Zhongda Hospital, School of Medicine, Southeast University, 210009 Nanjing, Jiangsu, China; ^8^Communist Youth League Committee, Fuwai Hospital, National Center for Cardiovascular Diseases, Fuwai Hospital, Chinese Academy of Medical Sciences, and Peking Union Medical College, 100037 Beijing, China; ^9^Department of Cardiac Surgery, Peking University People's Hospital, Peking University, 100044 Beijing, China

**Keywords:** Global Burden of Disease, ischemic heart disease, APC analysis, net drift, local drift

## Abstract

**Background:**

Ischemic heart disease (IHD) is the leading cause of mortality and disability worldwide. This study aimed to investigate global trends in IHD mortality across 204 countries and territories over the past 30 years and explore the influence of age, period, birth, and cohort effects on mortality.

**Methods:**

IHD mortality data were retrieved from the Global Burden of Disease (GBD) 2019 study. Temporal trends in the number of deaths, all-age mortality rates, and age-standardized mortality rates were assessed across countries grouped by sociodemographic index (SDI) quintiles. To quantify changes over time, we fitted age–period–cohort (APC) models and derived overall annual percentage changes (net drift) and age-specific annual percentage changes (local drift). The APC model was then used to distinguish the independent effects of age, period, birth, and cohort on IHD mortality trends.

**Results:**

The annual global IHD deaths increased from 5.70 million (95% uncertainty interval (UI): 5.41–5.90) to 9.14 million between 1990 and 2019 (95% UI: 8.40–9.74). All-age mortality rates also rose significantly, with a notable shift in deaths toward older populations (≥70 years). The global net drift in IHD mortality declined by 1.10% annually (95% confidence interval (CI): –1.17% to –1.04%), with high-SDI countries experiencing the greatest decline (–2.84%, 95% CI: –3.05% to –2.64%). Age, period, and birth cohort effects manifested a general declining trend. The largest positive net drift was observed in the Philippines (3.60%, 95% CI: 3.33%–3.86%). Key global risk factors included hypertension, elevated low-density lipoprotein cholesterol, ambient particulate matter pollution, and smoking. However, low temperatures were the leading environmental risk factor in high-SDI countries.

**Conclusions:**

From 1990 to 2019, the global burden and temporal trends for IHD mortality varied substantially across SDI quintiles, sex, geographic regions, and countries. These disparities underscore the need for region-specific, risk-differentiated, and cost-effective interventions to prevent and manage IHD. Moreover, strengthening primary healthcare, improving health system responsiveness, and enhancing health promotion and prevention efforts are critical, especially in regions where IHD mortality remains stable or is increasing.

## 1. Introduction

Ischemic heart disease (IHD) remains the leading cause of mortality and is also a major contributor to disability worldwide. In 2019, IHD accounted for 9.14 million deaths, 34.40 million years lived with disability (YLDs), and 182 million disability-adjusted life years (DALYs) [1, 2]. Although IHD-related mortality has decreased in developed countries, it has steadily risen in most developing countries due to the growing prevalence of cardiovascular risk factors. These include obesity, poorly controlled hypertension, diabetes mellitus, tobacco use, and poor lipid profiles [3, 4]. Population-level data on temporal changes in IHD mortality is crucial for identifying risk factors and designing effective interventional strategies, particularly in settings with limited resources [4, 5]. Previous large-scale epidemiology studies have generated valuable insights into age-specific mortality patterns. However, they often failed to distinguish the effects of aging from those attributable to specific birth cohorts or periods [5]. Aside from chronological aging, cohort and period factors can independently influence IHD mortality and disability. These may reflect technological advances in percutaneous coronary intervention (PCI), which primarily benefit the population born after a certain period, or geopolitical and economic instabilities at particular times that affect healthcare availability and cardiovascular health outcomes [1, 2, 5, 6]. Without separating these factors, analyses may yield misleading inferences and ineffective policy decisions.

To address these knowledge gaps, the present study analyzed IHD mortality trends across 204 countries and territories from 1990 to 2019 using the Global Burden of Disease (GBD) 2019 database. An age–period–cohort (APC) modeling approach was employed to examine the independent influences of aging, birth cohorts, and periods on mortality patterns. These findings contribute to a more nuanced understanding of the global epidemiology of IHD and support the development of tailored prevention and management strategies. This work was conducted within the GBD Collaborator Network and in accordance with GBD study protocols (Contact ID: 0034o00001nHH4NAAW).

## 2. Methods

### 2.1 Overview of GBD 2019

The GBD 2019 study provides comprehensive descriptive epidemiology for 369 diseases and injuries across 204 countries and regions during 1990–2019. Each death is attributed to a single underlying cause chosen from a mutually exclusive and collectively exhaustive list of diseases and injuries. The GBD 2019 study team obtained ethics approval from the University of Washington Institutional Review Board Committee. Detailed GBD protocols and all data are available online and can be requested through the EPHI-IHME Office (https://www.ncbi.nlm.nih.gov/pmc/articles/PMC9416661/). The GBD 2019 data are publicly accessible and contain no identifiable personal information (https://www.ncbi.nlm.nih.gov/pmc/articles/PMC9897059/).

### 2.2 Data Sources

We conducted secondary analysis of IHD mortality data from the GBD 2019 study, which can be accessed via the Global Health Data Exchange (GHDx) website (http://ghdx.healthdata.org/). This is a public database platform supported by researchers from 162 countries and territories since 1990. The GBD project continuously updates its disease lists, data sources, and methods [5]. GBD 2019 provides comprehensive mortality estimates from 1990 to 2019 at precise geographical locations, encompassing 204 countries and territories [5]. Cause of death (CoD) is coded using the International Classification of Disease, 10th Revision (ICD-10). IHD was defined in this study as ICD-10 codes I20–I25. GBD 2019 imported data from vital registration systems and verbal autopsy studies, and incorporated new dietary covariates into the existing standard Cause of Death Ensemble modeling (CODEm) approach. Detailed methodological descriptions can be found in Methodology Appendix 1 [5].

### 2.3 Temporal Trend Analysis

We employed crude and age-standardized mortality rates (ASMRs) to quantify the global burden of IHD deaths. Mortality rates were expressed as deaths per 100,000 person-years. Age standardization was performed using the GBD 2019 world population age standards [5]. We also calculated mortality ratios by comparing mortality rates to a mid-period reference. Successive birth cohorts (1895–2004, at 10-year intervals) were examined to explore temporal patterns. Additionally, we assessed the impact of 26 risk factors for IHD provided in GBD 2019 on changes in ASMRs.

### 2.4 Regional Trend Analysis

GBD 2019 incorporated the socio-demographic index (SDI) in its analysis. This composite socio-economic development indicator is based on income per capital, average years of schooling, and fertility in females aged <25 years. Higher SDI values denote greater socio-economic development. Although GBD 2019 scaled the SDI value from 0 to 100, we utilized the earlier GBD 2017 SDI scale that ranged from 0 to 1. All countries were categorized into five SDI groupings (high, high-middle, middle, low-middle, and low) according to their SDI quintiles.

### 2.5 APC Model Analysis

We implemented an APC model to disentangle the effects of age, period, and birth cohort on IHD mortality trends. APC model analysis is employed frequently in the descriptive epidemiology of chronic diseases. It enables examination of the individual effects of biological aging, historical periods, and generational cohorts on the disease rate. Moreover, it separates the contributions of technological and social factors from age-associated biological influences [6]. We used the APC model in R (version 3.6.3, R Foundation for Statistical Computing, Vienna, Austria), as described previously [7]. In a typical APC model, age and period are divided into equal time intervals. In this study, we set 16 age groups from 15 to 94 years in 5-year increments (15–19, …, 90–94 years). We also set 11 birth cohorts in 10-year increments (1895–1904, …, 1995–2004), with the mid-period (1945–1954) set as the reference. Period cohorts were defined as 6 five-year calendar periods (1990–1994, …, 2015–2019), with the reference period being 2000–2004. Using unconstrained parameters, we estimated the APC effects while mitigating the standard identification problem [6, 7].

The two most important APC parameters were net drift and local drift [7]. Net drift is defined as the overall annual percentage change in the age-standardized mortality rate across the study period, reflecting the combined time trend from period and cohort effects that is common to all ages. Local drift is defined as the age-specific annual percentage change in mortality over time for each age group [7]. The U.S. Healthy People 2020 Final Progress Table defines an objective that has moved <10% relative to its baseline over a 10-year period as “Little or No Detectable Change”, whereas an objective that has moved 10% or more is defined as “Got Worse or Improved” [8]. Therefore, a net or local drift in the present study of ±1% per year or more was considered substantial, and approximates to changes of ±10%, ±18%, and ±26% in fitted rates over 10-, 20-, and 30-years, respectively. APC model outputs also included fitted longitudinal age-specific rates adjusted for period deviations (representing age effects), and period- and cohort-specific relative risks (reflecting period and cohort effects). Period and cohort rate ratio curves incorporated the entire value of the net drift [7].

### 2.6 Statistical Analysis

Mortality data were presented as absolute numbers, all-age mortality rates, and ASMRs with 95% uncertainty intervals (UIs). Percentage changes were calculated as ([new value – old value] / old value) ×100. Relative risks (RRs) were expressed as the ratios of age-specific rates for a given period (or birth cohort) to the corresponding rate in the designated reference period (or cohort). Trends in annual percentage change derived from the APC model were evaluated using Wald chi-square tests. All tests were two-sided, and statistical significance was set at *p* < 0.05. The proportion of deaths refers to the percentage of all IHD deaths in the corresponding group. Rates were depicted as per 100,000 person-years. All analyses were performed in R (version 3.6.3).

## 3. Results

### 3.1 Overall Temporal Trends in IHD Mortality Across SDI Groups

Trends in population size, total number of IHD deaths, all-age mortality rates, ASMR, and the net drift in mortality by SDI quintiles from 1990 to 2019 are shown in Table 1, **Supplementary Table 1** and **Supplementary Figs. 1,2**. Globally, annual IHD deaths increased from 5.70 million (95% UI: 5.41–5.90) in 1990 to 9.14 million (95% UI: 8.40–9.74) in 2019, reflecting a 60.43% rise. During the same period, the world population grew by approximately 45%, from 5.35 billion (95% UI: 5.23–5.46) to 7.74 billion (95% UI: 7.49–7.93).

**Table 1.** **Trends in IHD mortality stratified by SDI quintile, 1990–2019**.

	Number of deaths 1990*, n × 1000	Number of deaths 2019, n × 1000	Percent change of deaths (1990–2019)	ASMR 1990 (per 100,000)	ASMR 2019 (per 100,000)	Percent change of ASMR 1990–2019
Global	5695.89 (5405.19, 5895.40)	9137.79 (8395.68, 9743.55)	60.43 (50.23, 69.14)	170.45 (159.61, 176.94)	117.95 (107.83, 125.92)	–30.80 (–34.83, –27.17)
High-SDI	1688.79 (1572.96, 1744.99)	1447.27 (1270.02, 1553.84)	–14.30 (–19.35, –9.59)	162.39 (150.62, 168.15)	67.10 (60.07, 71.54)	–58.68 (–60.30, –56.69)
High-middle SDI	1870.95 (1782.68, 1923.52)	2658.29 (2411.87, 2832.48)	42.08 (32.95, 50.53)	209.20 (196.25, 216.23)	135.41 (122.68, 144.44)	–35.28 (–39.01, –31.69)
Middle-SDI	1151.13 (1087.98, 1217.99)	2824.55 (2576.47, 3047.02)	145.37 (122.94, 167.00)	143.11 (133.18, 152.13)	134.12 (121.51, 145.22)	–6.28 (–14.41, 1.84)
Low-middle SDI	712.69 (654.08, 773.46)	1646.06 (1488.07, 1801.77)	130.96 (104.70, 156.95)	144.21 (132.05, 156.58)	136.59 (122.96, 149.50)	–5.28 (–15.25, 5.09)
Low-SDI	269.14 (240.68, 301.92)	556.60 (495.17, 627.06)	106.81 (78.43, 134.17)	139.20 (124.00, 156.79)	127.99 (113.13, 143.9)	–8.05 (–20.59, 3.17)

ASMR, age-standardized mortality rate; SDI, socio-demographic index; IHD, ischemic heart disease. **Notes**: The ASMR is derived by direct standardization to the Global Burden of Disease (GBD) 2019 global standard population; A leading “–” indicates decrease in 2019 relative to 1990. * Parentheses indicate 95% uncertainty intervals for all estimates.

Between 1990 and 2019, high-SDI countries experienced a 14.30% (95% UI: –19.35% to –9.59%) reduction in IHD-related deaths. In contrast, marked increases occurred in other SDI groups: 42.08% (95% UI: 32.95%–50.53%) in high-middle SDI countries, 145.37% (95% UI: 122.94%–167.00%) in middle-SDI countries, 130.96% (95% UI: 104.70%–156.95%) in low-middle SDI countries, and 106.81% (95% UI: 78.43%–134.17%) in low-SDI countries. Consequently, the proportion of global IHD deaths in high-SDI countries declined from 29.65% in 1990 to 15.84% in 2019, whereas in middle-SDI countries they accounted for a substantial 30.91% of total IHD deaths in 2019.

All-age mortality rates reflected similar patterns. The global all-age mortality rate increased from 106.70 (95% UI: 101.03–110.20) to 118.10 (95% UI: 108.51–125.93) per 100,000 person-years. Across SDI quintiles, only high-SDI and low-SDI countries saw decreases in all-age mortality. High-SDI countries demonstrated a –30.49% (95% UI: –34.58% to –26.67%) reduction, while low-SDI countries exhibited a modest –3.23% (95% UI: –16.51% to 9.58%) decrease. In contrast, high-middle SDI countries showed the highest all-age mortality rate, reaching 185.84 (95% UI: 168.61–198.02) per 100,000 person-years in 2019.

ASMR, however, declined consistently across all SDI groups over the past 30 years. Globally, ASMR decreased from 170.45 (95% UI: 159.61–176.94) to 117.95 (95% UI: 107.83–125.92) per 100,000 person-years. High-SDI countries experienced the largest relative reduction in ASMR of –58.68% (95% UI: –60.30% to –56.69%), followed by –35.28% in high-middle SDI countries (95% UI: –39.01% to –31.69%). In 2019, high-SDI countries recorded the lowest ASMR of 67.10 (95% UI: 60.07–71.54) per 100,000 person-years. Other SDI groups had similar ASMRs, ranging from 144.21 (95% UI: 132.05–156.58) to 136.59 (95% UI: 122.96–149.50) per 100,000 person-years.

Net drift estimates from the APC model mirrored the ASMR trends. From 1990 to 2019, the global net drift in annual mortality declined by –1.10% (95% CI: –1.17% to –1.04%) per year. High-SDI countries witnessed the steepest decline of –2.84% (95% CI: –3.05% to –2.64%), while low-middle SDI countries showed the smallest decrease of –0.26% (95% CI: –0.38% to –0.15%).

### 3.2 National Trends in IHD Mortality (1990–2019)

National-level trends varied significantly, and being in a higher SDI quintile did not always mean lower IHD deaths (**Supplementary Fig. 3** and **Supplementary Table 2**). In 2019, 15 countries and regions recorded at least 100,000 IHD deaths, with the highest being in China (1,874,000, 95% UI: 1,612,000–2,132,000), India (1,519,000, 95% UI: 1,311,000–1,746,000), the Russian Federation (563,000, 95% UI: 489,000–633,000), the United States (558,000, 95% UI: 497,000–594,000), and Ukraine (326,000, 95% UI: 285,000–372,000), collectively accounting for 52.90% of global IHD deaths.

Between 1990 and 2019, disturbingly high increases in all-age IHD mortality were observed in the Northern Mariana Islands (267.09%, 95% UI: 189.90%–357.40%), Philippines (169.25%, 95% UI: 96.66%–227.81%), Timor-Leste (164.87%, 95% UI: 98.31%–242.31%), Albania (158.39%, 95% UI: 102.31%–226.40%), and China (156.61%, 95% UI: 115.61%–205.68%). Notably, Uzbekistan was the only country in which ASMR increased by more than 100% (119.01%, 95% UI: 96.32%–143.33%).

Regarding net drifts, 64 countries exhibited stable or rising mortality trends (net drift ≥–0.50%), despite global declines. Nine countries showed net drift of ≥1%, including the Philippines (3.60%, 95% CI: 3.33%–3.86%), Lesotho (2.63%, 95% CI: 2.02%–3.25%), Uzbekistan (1.99%, 95% CI: 1.74%–2.24%), Mozambique (1.78%, 95% CI: 1.58%–1.99%), Zimbabwe (1.61%, 95% CI: 1.35%–1.86%), Dominican Republic (1.51%, 95% CI: 1.30%–1.73%), Timor-Leste (1.48%, 95% CI: 0.77%–2.19%), Kenya (1.37%, 95% CI: 1.15%–1.58%), and Guinea (1.17%, 95% CI: 0.90%–1.45%). A 1% annual increase in net drift predicts increases in the mortality rate of 10%, 18%, and 26% over the next 10-, 20-, and 30-years, respectively. Targeted interventions are therefore urgently needed in these regions.

### 3.3 Age- and Sex-Specific Temporal Trends in IHD Mortality

**Fig. 1.**
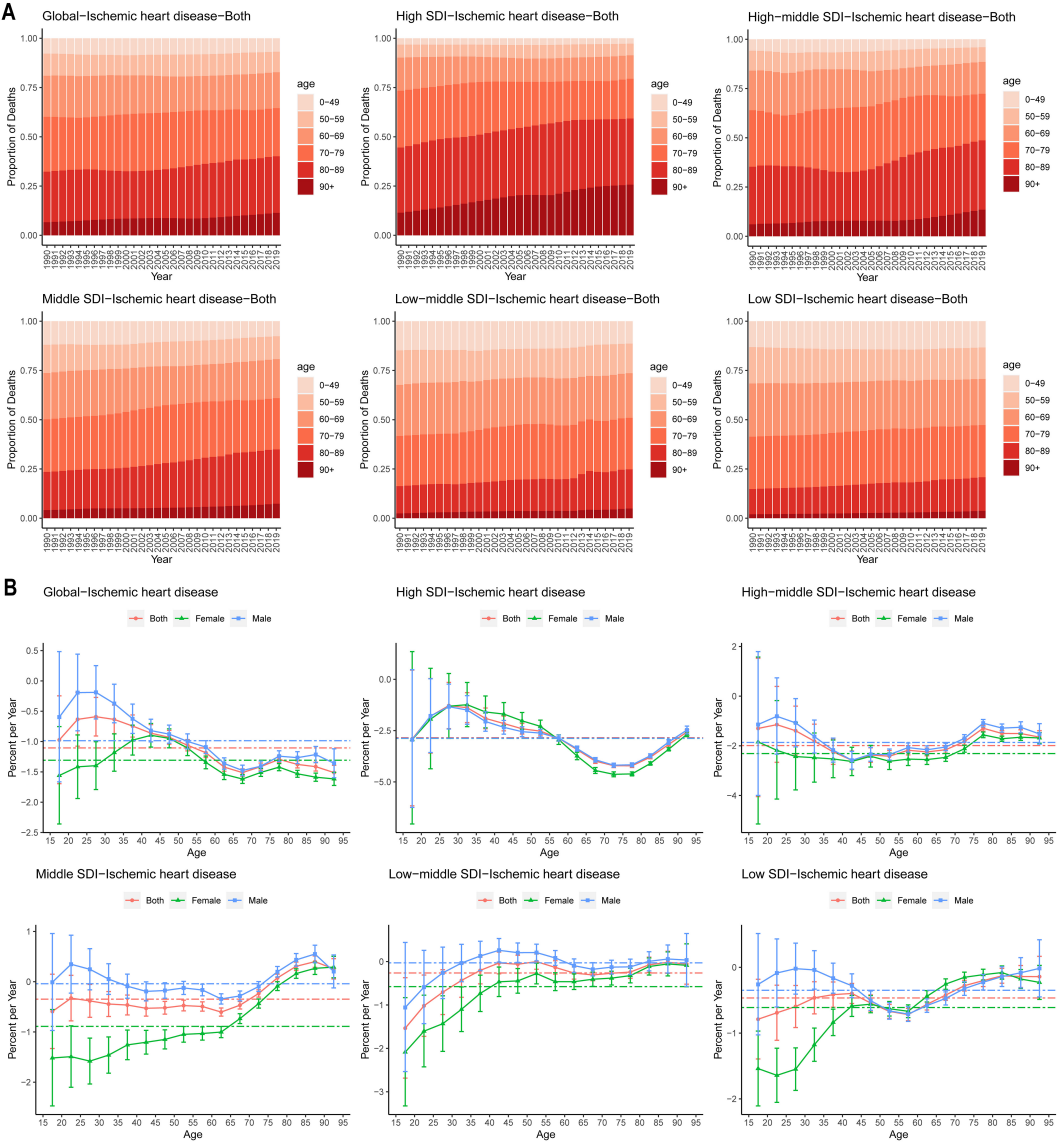
**Local drifts in IHD mortality, and age distribution of deaths by SDI quintiles, 1990-2019**. (A) Temporal change in the age distribution of IHD, shown as the proportion of deaths in each age group (0–49, 50–59, 60–69, 70–79, 80–89, 90+ years). (B) Local drifts in IHD mortality, estimated from APC models, for 16 age groups with 5-year increments (15–19 to 90–94 years). The dots and vertical lines indicate the annual percentage change in mortality (% per year) and the corresponding 95% CIs. APC, age–period-cohort; IHD, ischemic heart disease; SDI, socio-demographic index.

From 1990 to 2019, IHD mortality shifted towards older populations (≥70 years), particularly in high- and high-middle SDI regions (Fig. 1A). In these areas, women aged ≥70 years accounted for over 75% of all IHD deaths in females. This proportion was higher than that observed among men in the same age group. In contrast, more than half of IHD deaths in low-middle and low-SDI countries occurred in individuals aged <70 years, potentially foreshadowing increased future mortality (**Supplementary Fig. 4**).

APC modeling by age group (local drift) revealed global declines in IHD mortality for all ages, with the greatest reduction of –4.63% (95% CI: –4.75% to –4.51%) observed among older women aged 70–74 years in high-SDI countries (Fig. 1B). No significant sex differences were observed in high- and high-middle SDI countries, indicating uniform improvements in both sexes. However, in the middle-, low-middle, and low-SDI countries, nearly 50% of age groups showed stable or even increasing mortality rates. Interestingly, female mortality in these regions improved more rapidly than male mortality.

### 3.4 Age, Period, and Cohort Effects on IHD Mortality Across SDI Groups

**Fig. 2.**
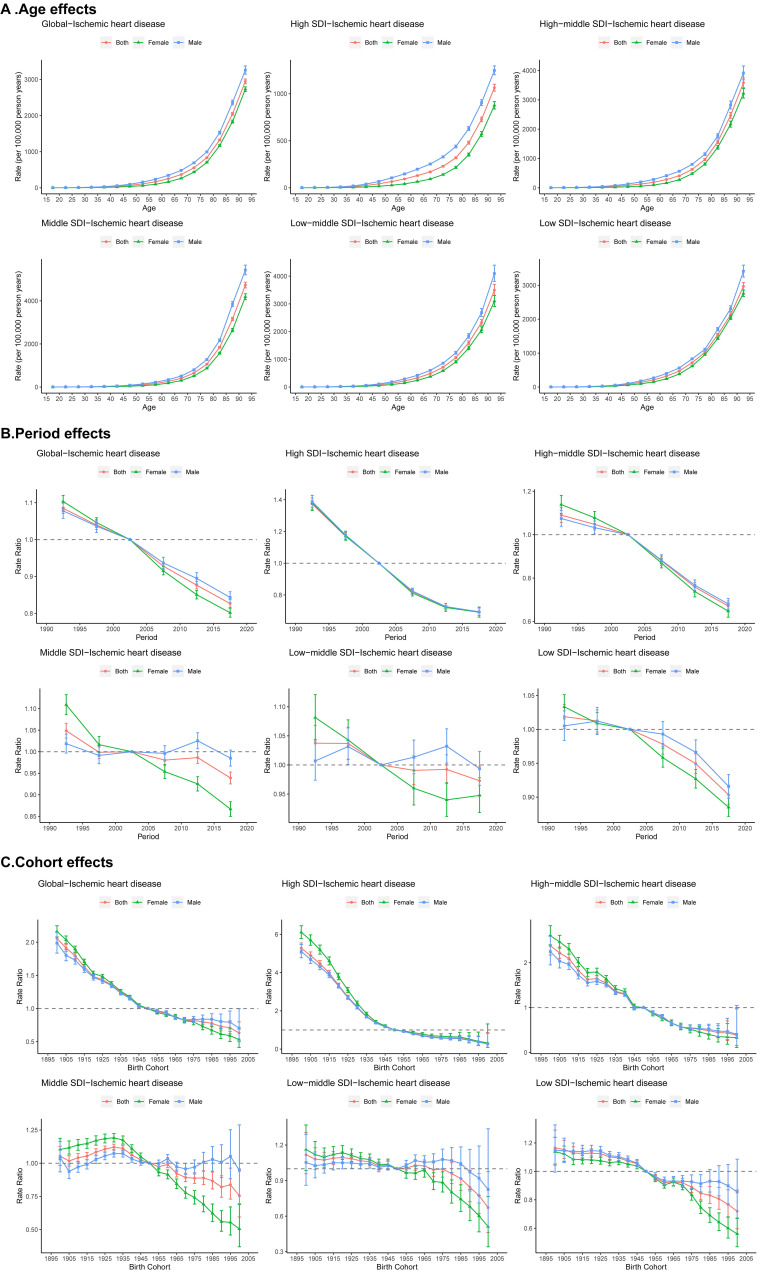
**Age, period, and cohort effects on IHD mortality by SDI quintiles**. (A) The fitted longitudinal curves show the age effects on mortality, adjusted for period effects. (B) Period effects on mortality are shown by the ratio of age-specific mortality rates, from 1990–1994 to 2015–2019, with 2000–2004 being the reference period. (C) Cohort effects on mortality are shown by the ratio of age-specific mortality rates, from the 1895 cohort to the 2004 cohort, with the 1945–1954 cohort being the reference. The mortality rates or RRs and their corresponding 95% CIs are presented. IHD, ischemic heart disease; SDI, socio-demographic index; RR, rate ratio.

APC models separated the individual contributions of age, period, and cohort effects on IHD mortality trends (Fig. 2). Advancing age was consistently associated with increased mortality risk. Significant sex differences emerged after the 20–24 age group (RR = 1.32, z = 3.95, *p* < 0.001) and biggest difference at 45–49 age group (RR = 2.67, z = 46.16, *p* < 0.001), and peaking difference in rates occurs in the 90–94 age group (Fig. 2A). A general decrease in IHD mortality risk was observed across all SDI quintiles as time progressed. However, male populations in middle- and low-middle SDI countries experienced fluctuations above a relative risk of one after 2005 (Fig. 2B). Cohort effects manifested as long-term reductions in most settings, except for persistently elevated risk (RR >1) in males from middle-SDI countries until the 2000 birth cohort (RR = 0.95, 95% CI: 0.70–1.29), and in low-middle SDI countries until the 1990 cohort (RR = 0.97, 95% CI: 0.81–1.16) (Fig. 2C). These findings indicate delayed improvement in IHD mortality among men in middle- and low-middle SDI regions.

### 3.5 Age, Period, and Cohort Effects in Selected Countries

**Fig. 3.**
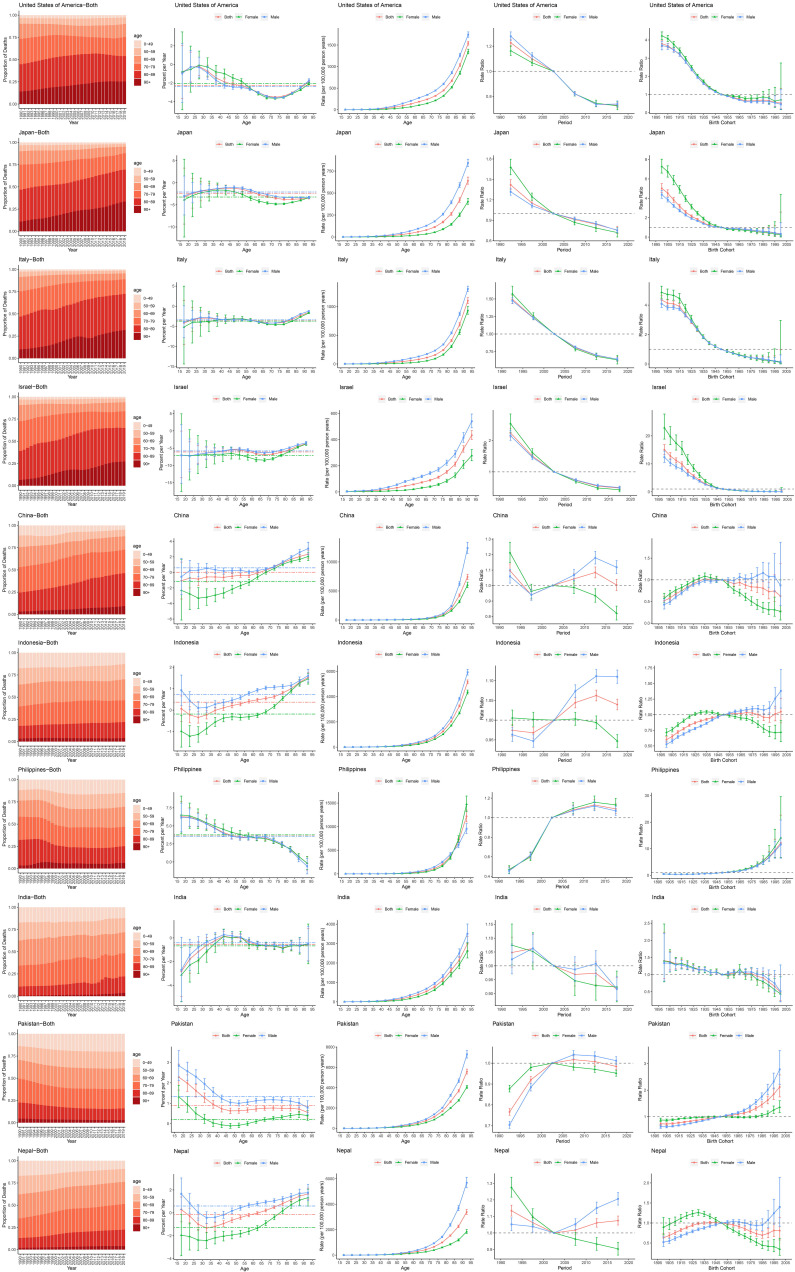
**APC effects on mortality in selected countries**. APC, age–period–cohort.

Fig. 3 illustrates APC effects in 10 representative countries spanning the SDI spectrum over the past 30 years. High-SDI countries, such as the United States and Japan, exhibited pronounced shifts in IHD mortality towards older age groups (≥70 years), and consistent reductions in mortality rates across periods and cohorts. High-middle SDI countries, such as Italy and Israel, showed similar patterns. Among middle-SDI countries, China exhibited a trend similar to high- and high-middle SDI countries, while IHD mortality in Indonesia remained more evenly distributed across age groups. Middle-SDI countries exhibited similar trends in local drift, age, and period effects, although sex differences were smaller compared to high- and high-middle SDI countries. Although both countries recorded overall declines, period effects were generally higher for men. Cohort effects in China followed an inverted U-shape, while Indonesia exhibited similar patterns but mainly in females, suggesting heterogeneous improvements.

Conversely, in low-middle and low-SDI countries, no substantial shift in IHD mortality towards older age groups was observed. The trend even reversed in some cases. For example, local drift trends were consistently down in the Philippines and Pakistan, but up in India and Nepal, indicating rising mortality rates. Birth cohort effects varied widely: the Philippines and Pakistan exhibited upward trends, whereas Nepal showed an “inverted U-shaped” pattern, and India demonstrated a downward trend.

### 3.6 Risk Factor Analysis

**Fig. 4.**
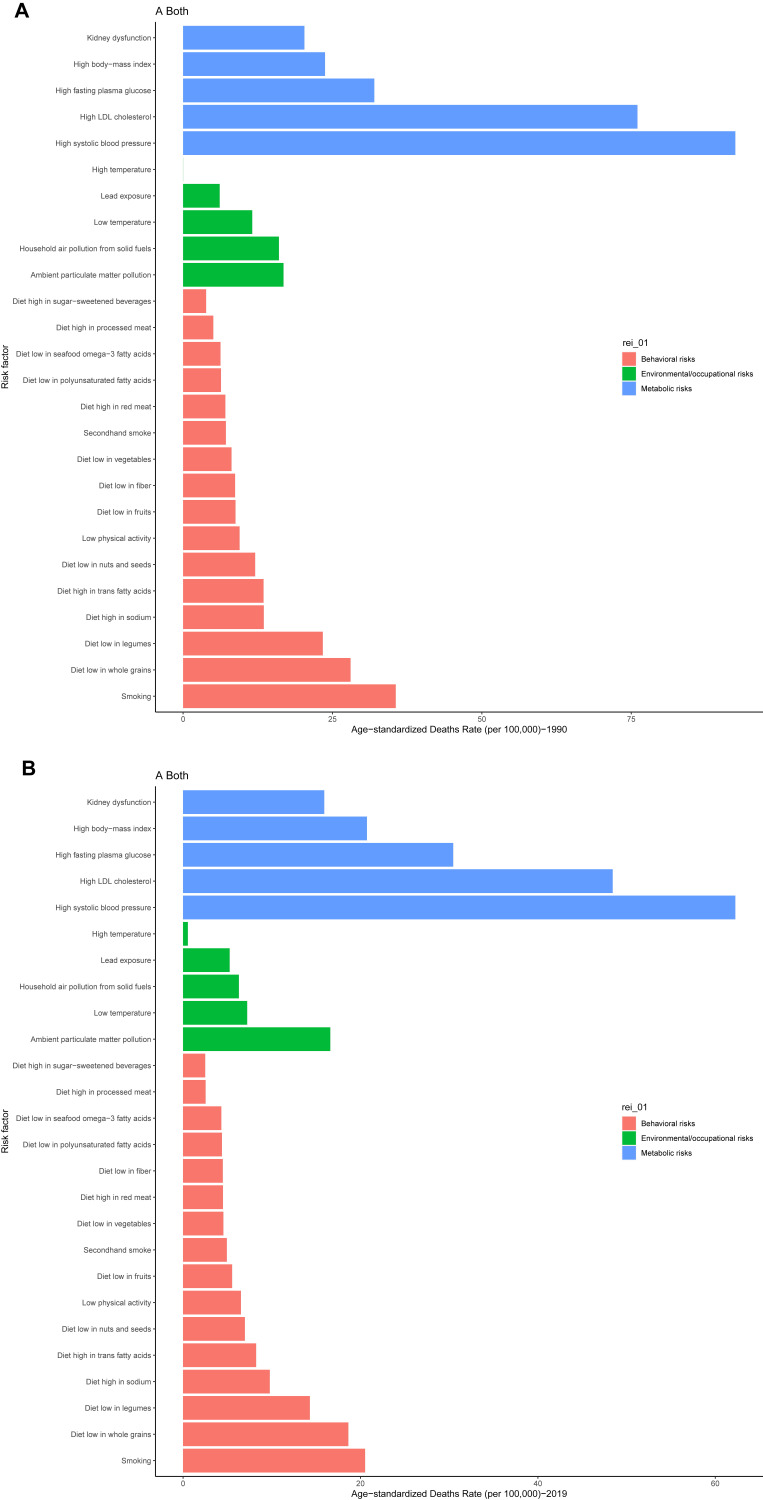
**Major risk factors for global age-standardized IHD death, 1990–2019**. (A) Age-standardized mortalities by different risk factors in 1990. (B) Age-standardized mortalities by different risk factors in 2019. Every factor represents a category of risk factor. IHD, ischemic heart disease; LDL, low-density lipoprotein.

Fig. 4 and **Supplementary Fig. 5** show risk factor distributions and their contribution to IHD mortality or DALYs across SDI countries. Over the past 30 years, metabolic factors (notably high low-density lipoprotein (LDL) cholesterol and hypertension) have remained the dominant contributors, despite overall declines in ASMR. Environmental risks, including ambient particulate matter pollution and low temperatures, declined worldwide but remained substantial. Low temperatures emerged as the leading environmental risk in high-SDI countries, while ambient pollution from particulate matter stood out as a key environmental concern in middle- and low-SDI countries. Behavioral factors, such as smoking, unhealthy dietary habits, and low physical activity, continue to contribute to mortality. However, smoking-related mortality declined in high- and high-middle SDI quintiles. Middle- and low-middle SDI countries saw mixed progress, with improved control of some behavioral risks, but sustained burdens of metabolic risks.

## 4. Discussion

Although IHD remains a leading cause of death globally, sharp disparities persist across SDI groups and countries. These are influenced by age structure, socioeconomic conditions, healthcare availability, and risk factor profiles.

Total deaths and all-age mortality rates have generally increased over the past 30 years, except in high-SDI countries. Half the total deaths were in the 60–89-year-old population, while the mortality rate among people aged >90 years increased significantly, especially in high-SDI nations. However, ASMR and net drift decreased worldwide. Local drifts showed the same pattern, except for the 75–79-year age group in middle-SDI countries, and males aged 30–64 years in low-middle-SDI countries.

In high-SDI countries, the increased proportion of deaths in people aged ≥70 years may reflect broader application of secondary prevention measures. Accordingly, policies should emphasize the comprehensive management of working-age men in low-middle SDI countries [9].

APC analyses highlight the importance of distinguishing between age, period, and cohort effects when interpreting mortality trends [6]. Age effects primarily reflect aging and the accumulation of cardiometabolic risk for IHD. Period effects capture time-specific influences on IHD mortality, including improvements in health-system capacity, broader detection and control of high-risk conditions such as hypertension, dissemination of medical technologies, period-characteristic environmental exposures, and major policy changes. Cohort effects represent intergenerational risk histories, such as the initiation or cessation of smoking, and early-life nutrition and education, that shape IHD risk later in life. The social circumstances from these APC statistic mappings across different SDI countries will inform the formulation of policies such as primary care-based hypertension programs, and coverage of essential cardiovascular medicines, adoption of clean energy, and the timely regulation of tobacco consumption.

While high-SDI countries achieved substantial improvements across all age groups, slower progress or even rising mortality was seen in middle-, low-middle, and low-SDI countries. Females experienced greater reductions in mortality than males in lower-SDI settings, underscoring potential sex-specific barriers and opportunities. The findings suggest that while “male-friendly” primary healthcare and health promotion strategies may need to be established, efforts should also focus on maintaining and expanding clean energy and indoor air quality control to continue the benefits for women. Previous research has shown that women generally seek medical treatment and undergo primary prevention or screening at higher rates than men. This is especially the case for health awareness, treatment, and control of hypertension, thus contributing to the earlier and more sustained management of metabolic risk factors [10]. The burden of IHD and other cardiopulmonary diseases caused by indoor air pollution is severe in households using solid fuels, where women and children experience higher exposure. Moreover, several studies have indicated that women may have a greater risk of IHD from ambient particulate matter pollution than men [11, 12, 13]. Therefore, the decline in female IHD may become even more pronounced with the adoption of clean energy and subsequent improvement in air quality.

Globally, the principal risk factors for IHD remain hypertension, smoking, LDL-C, diabetes, and ambient particulate matter pollution. In low-middle SDI regions, the local drift for men aged 30–64 years showed an increase rather than decrease, consistent with a “working-age gap”, and may reflect insufficient control of the modifiable risks [9]. The use rate of secondary prevention drugs such as statins, antiplatelet agents and antihypertensive drugs is significantly lower in low-income countries. This aligns with the trend in low- and middle-income and low-SDI countries, where the net drift does not improve or even worsens, indicating poor treatment accessibility and patient compliance in these regions [14]. With industrialization and urbanization, environmental exposures have shifted from household air pollution to ambient particulate matter, especially in middle-, low-middle, and low-SDI regions. Meanwhile, this study found that “low temperature” effects were more prominent in high-SDI countries. We therefore recommend implementation of universal policies to reduce ambient particulate pollution, as well as a specific focus on winter protection, building insulation, and reliable heating in high-SDI countries [15].

The problems of some typical countries may reflect common deficiencies in the corresponding SDI-level group, as well as policy-oriented directions. However, intervention measures need to be implemented in accordance with specific national conditions. With regard to high-SDI regions, for example, targeted interventions are needed for the aging population in the United States, and for high alcohol consumption in Russia [16, 17]. Populous middle-SDI nations like China, India, and Indonesia contribute enormously to the global death toll and experienced marked increases in ASMRs. Positive net drifts were observed in these countries due to poorly controlled metabolic risk factors and epidemiological transitions, including population expansion, aging societies, and rapid urbanization [18, 19, 20, 21]. Inadequate access to high-quality healthcare also played a crucial role, as over half the IHD-related deaths in India occurred in individuals with pre-existing IHD who received no medication [19]. Among low-middle and low-SDI countries, the Philippines exemplifies the urgent need for context-specific interventions. It had the largest increase in death toll, all-age mortality rates, and very high net drift.

Various public health interventions are being rolled out worldwide to reduce IHD [22]. Since 2017, the World Health Organization (WHO) has collaborated with the Guam government to combat smoking by imposing tobacco taxes and improving tobacco regulations to meet WHO standards [23]. WHO has also developed practical disease surveillance and management tools, including the WHO Stepwise Approach to Noncommunicable Disease Risk Factor Surveillance (WHO STEPS), and the WHO Package of Essential Noncommunicable Disease Interventions (WHO PEN). These tools integrate simple methodologies and clinical protocols to measure and manage cardiovascular disease (CVD) in primary health care (PHC) settings [24, 25]. Such interventions have been implemented in resource-limited nations like Ukraine, Uzbekistan, and Tajikistan, significantly enhancing the quantitative and qualitative aspects of primary care [22, 26, 27, 28]. Healthcare reforms promoting PHC have also been introduced in nations severely affected by IHD mortality, such as Ukraine and the Philippines [26, 29]. However, mixed results have been reported due to complex implementation barriers, unsustainable development, and inadequate evaluation mechanisms [30]. Interventions must be tailored to local contexts, incorporating WHO guidelines, cost-effective primary care approaches, and robust implementation research frameworks. A promising alternative is the Consolidated Framework for Implementation Research (CFIR), which offers potential value in overcoming these challenges and ensuring the effective management of IHD risk factors in resource-limited settings [22]. To enhance policy comprehension, we also propose translating the APC net drift threshold of ±1% per year into illustrative 10-, 20-, and 30-year cumulative changes (±10%, ±18%, and ±26%, respectively). These bands can define early-warning thresholds and support a tiered ranking for countries by intervention priority.

### Limitations

This study has several limitations. First, GBD 2019 excludes data before 1990, which limits the assessment of younger cohorts. Second, information on CVD morbidity in low- and middle-income nations has still not been completed to GBD data standards. Third, this analysis did not focus on non-fatal outcomes, which are critical consequences of IHD. Finally, this study utilized GBD 2019 data rather than the most recent GBD database, which extends into the COVID-19 era. The rationale was to avoid the impacts of the COVID-19 pandemic. Previous studies have indicated that COVID-19 influenced the post-2019 cardiovascular mortality risk, altered the mortality patterns, and changed the cause-of-death attribution [31, 32]. By restricting our analyses to the GBD 2019 cycle, we minimized pandemic-related period shocks, methodological complexities, and challenges in longitudinal comparability. Consequently, our findings reflect pre-pandemic trends. Nevertheless, it is likely that COVID-19 reshaped cardiovascular mortality dynamics in the subsequent years, and our future work will aim to replicate and extend the current analyses using post-2019 GBD data to capture potential pandemic-related shifts.

## 5. Conclusion

Over the past 30 years, age-standardized mortality rates for CVD have declined globally. Nevertheless, significant disparities exist between the sexes and between different SDI nations. Contributing to these differences are population aging, persistent metabolic and environmental risks, and uneven access to healthcare. Targeted policies, appropriate resource allocation, and interventions that are focused on modifiable risk factors are essential to reduce the global burden of IHD and achieve equitable health outcomes.

## Availability of Data and Materials

The datasets generated and analyzed during the current study are available in the Global Health Data Exchange (http://ghdx.healthdata.org).

## Supplementary Material

Supplementary material associated with this article can be found, in the online version, at https://doi.org/10.31083/RCM45099.
