# Supplementary material for: Global, Regional, and National Time Trends in Mortality for Ischemic Heart Disease, 1990–2019: An Age-Period-Cohort Analysis for the Global Burden of Disease 2019 Study
Source: Rev Cardiovasc Med. 2025 Sep 30;26(9):45099. doi: 10.31083/RCM45099 (PMC12516774; doi:10.31083/RCM45099)
Supplement: Supplementary file 1 [file 2153-8174-26-9-45099-s1.zip › Supplementary Material.docx]

**Global, regional, and national time trends in mortality for ischemic heart disease, 1990-2019: an age-period-cohort analysis for the global burden of disease 2019 study**

[**Figure of Contents**](#_Toc31229)

[Supplementary Fig. 1. The all-age mortality rate in 2019 and percentage changes of the all-age mortality rate of IHD, 1990-2019 2](#_Toc8659)

[Supplementary Fig. 2. The age-standardized mortality rate in 2019 and percentage changes of ASR ,1990-2019 3](#_Toc2721)

[Supplementary Fig. 3. ASR and ASR changes in different SDI countries 4](#_Toc22456)

[Supplementary Fig. 4. Trends in the age distribution of death owing to IHD across countries and regions with different SDI for the entire population and for male and female populations, 1990-2019. 6](#_Toc8349)

[Supplementary Fig. 5. Major risk factors for age-standardized death of IHD across 5 SDI regions, 1990–2019. 12](#_Toc8544)

[**Table of Contents**](#_Toc2291)

[Supplementary Table 1. Trends in IHD mortality across Socio-demographic Index quintiles, 1990-2019 13](#_Toc24599)

[Supplementary Table 2. The APC analysis results of all 204 countries and regions. 15](#_Toc3784)

**Figure of Contents**


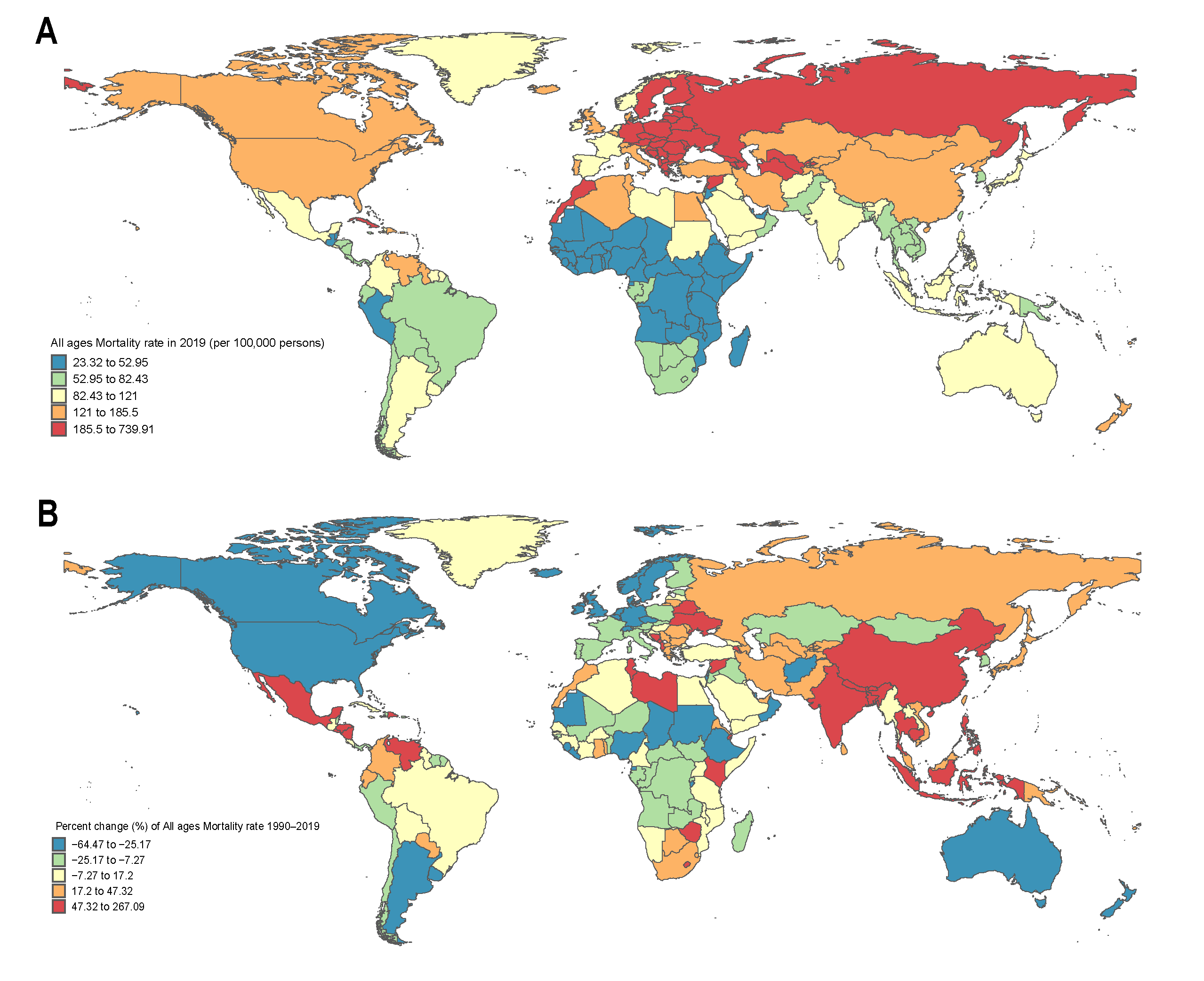


**Supplementary Fig. 1. The all-age mortality rate in 2019 and percentage changes of the all-age mortality rate of IHD, 1990-2019**

(A)The world map showed the all-age Mortalities in 2019 in different countries across the globe. (B)The world map depicted the percentage changes in all-age mortality rates globally in the past 30 years. IHD = Ischemic heart disease

**
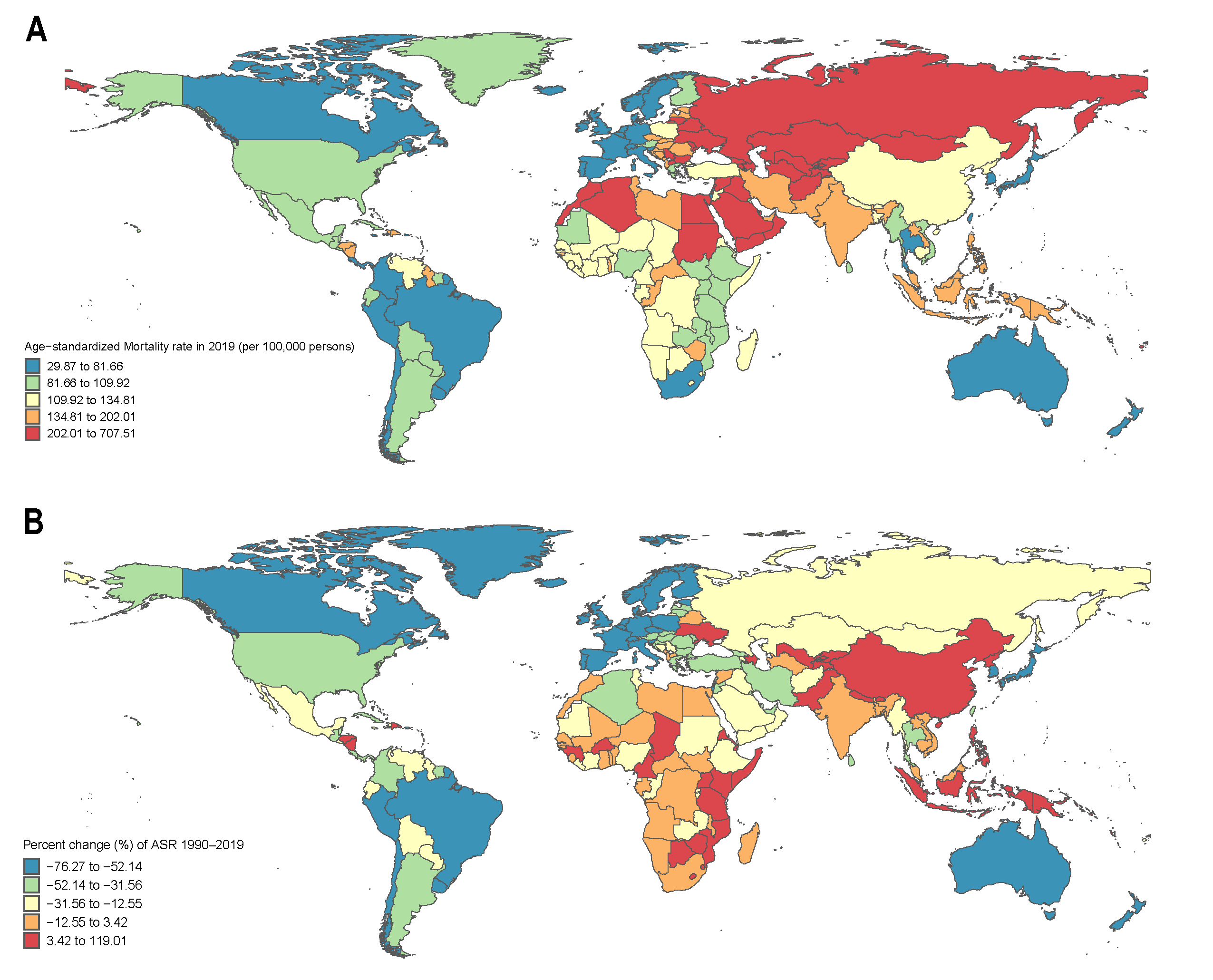
**

**Supplementary Fig. 2. The** **age-standardized mortality rate in 2019 and** **percentage changes of ASR ,1990-2019**

(A)The world map showed the age-standardized mortality rate in 2019 in different countries across the globe.(B)The world map depicted the percentage changes of ASR during 1990-2019 across the globe.IHD = Ischemic heart disease;ASR = age-standardized mortality rate.


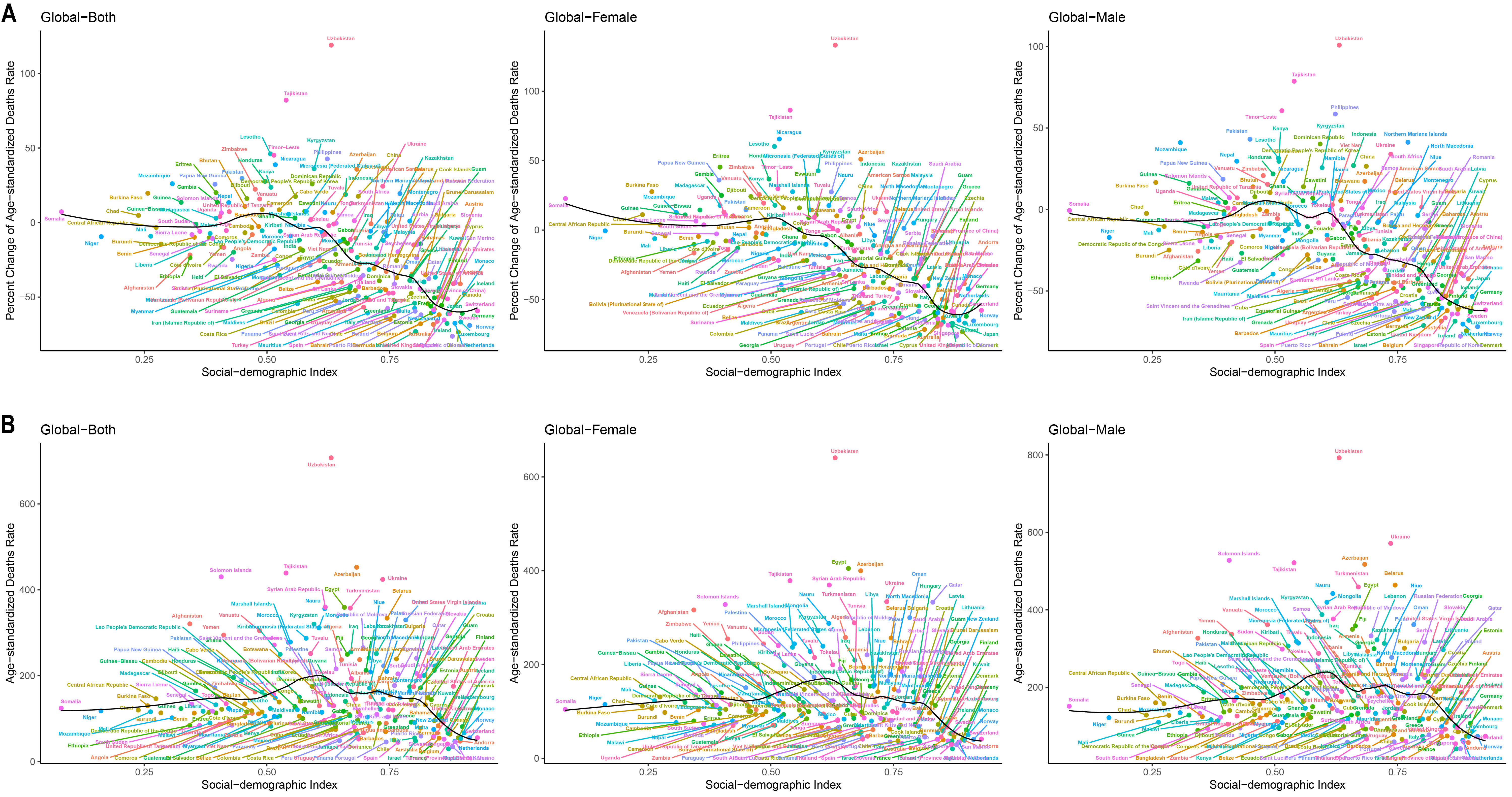


**Supplementary Fig. 3. ASR and ASR changes in different SDI countries**

(A)The relationship between the value of SDI and the 30-year percent change in ASR in both male and female populations globally. (B)The relationship between the value of SDI and ASR in 2019. SDI = Social-demographic index; ASR = age-standardized death rate. The X-axis represents the Social-demographic index, and the y-axis represents the percent change in the age-standardized rate. Every dot stands for a particular country.





**Supplementary Fig. 4. Trends in the age distribution of death owing to IHD across countries and regions with different SDI for the entire population and for male and female populations, 1990-2019.IHD = Ischemic heart disease; SDI=Socio-demographic Index.**


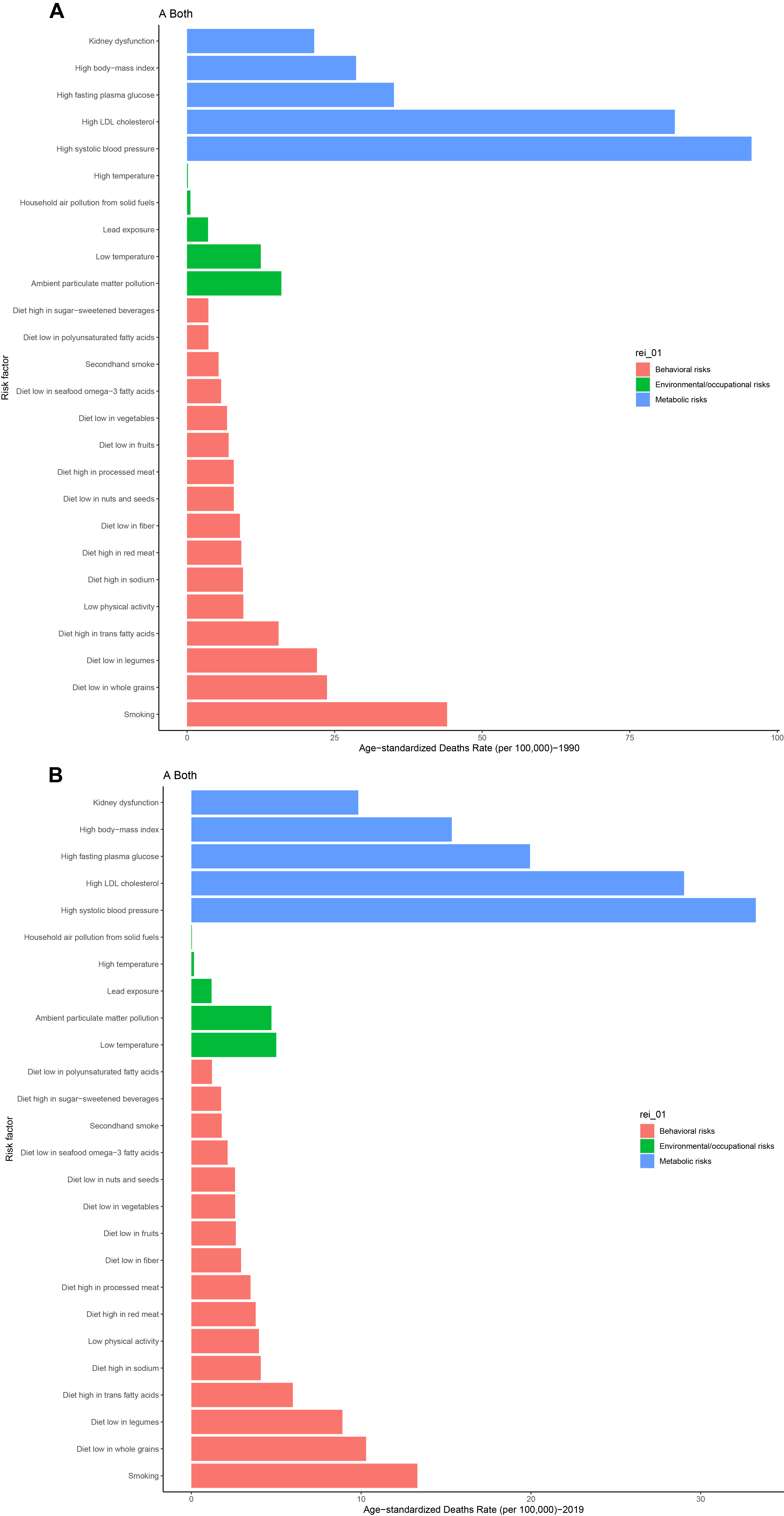














**Supplementary Fig. 5. Major risk factors for age-standardized death of IHD across 5 SDI regions, 1990–2019.**

(A)Age-standardized mortalities by different risk factors in high SDI countries in 1990. (B)Age-standardized mortalities by different risk factors in high SDI countries in 2019. (C)Age-standardized mortalities by different risk factors in high-middle SDI countries in 1990. (D)Age-standardized mortalities by different risk factors in high-middle SDI countries in 2019. (E)Age-standardized mortalities by different risk factors in middle SDI countries in 1990. (F)Age-standardized mortalities by different risk factors in middle SDI countries in 2019 Every factor represents a category of risk factor. (G)Age-standardized mortalities by different risk factors in low-middle SDI countries in 1990. (H)Age-standardized mortalities by different risk factors in low-middle SDI countries in 2019 Every factor represents a category of risk factor. (I)Age-standardized mortalities by different risk factors in low SDI countries in 1990. (J)Age-standardized mortalities by different risk factors in low SDI countries in 2019 Every factor represents a category of risk factor. IHD = Ischemic heart disease; SDI = Socio-demographic Index.

**Table of Contents**

**Supplementary Table 1. Trends in IHD mortality across Socio-demographic Index quintiles, 1990-2019**

|  | **Global** | | **High SDI** | | **High-middle SDI** | | **Middle SDI** | | **Low-middle SDI** | | **Low SDI** | |
| --- | --- | --- | --- | --- | --- | --- | --- | --- | --- | --- | --- | --- |
|  | **1990** | **2019** | **1990** | **2019** | **1990** | **2019** | **1990** | **2019** | **1990** | **2019** | **1990** | **2019** |
| **Population** | | | | | | | | | | | | |
| Number, n ×1,000,000 | 5350 (5239,5460) | 7737 (7483,7993) | 822 | 1013 | 1150 | 1430 | 1717 | 2397 | 1130 | 1764 | 528 | 1128 |
| Percentage of global,% | 100 | 100 | 15.40 | 13.10 | 21.50 | 18.50 | 32.10 | 39.60 | 21.10 | 22.80 | 9.90 | 14.60 |
| **Deaths** | | | | | | | | | | | | |
| Number*, n× 1,000 | 5695.89  (5405.19,5895.4) | 9137.79  (8395.68,9743.55) | 1688.79  (1572.96,1744.99) | 1447.27  (1270.02,1553.84) | 1870.95  (1782.68,1923.52) | 2658.29  (2411.87,2832.48) | 1151.13  (1087.98,1217.99) | 2824.55  (2576.47,3047.02) | 712.69  (654.08,773.46) | 1646.06  (1488.07,1801.77) | 269.14  (240.68,301.92) | 556.6  (495.17,627.06) |
| Percentage of global,% | 100 | 100 | 29.65 | 15.84 | 32.85 | 29.09 | 20.21 | 30.91 | 12.51 | 18.01 | 4.73 | 6.09 |
| Percent change of deaths  1990–2019,% | 60.43  (50.23,69.14) | | -14.3  (-19.35,-9.59) | | 42.08  (32.95,50.53) | | 145.37  (122.94,167.00) | | 130.96  (104.70,156.95) | | 106.81  (78.43,134.17) | |
| **All-age mortality rate** | | | | | | | | | | | | |
| Rate per 100,000 | 106.47  (101.03,110.2) | 118.1  (108.51,125.93) | 205.45  (191.35,212.28) | 142.82  (125.32,153.33) | 162.63  (154.96,167.20) | 185.84  (168.61,198.02) | 67.05  (63.37,70.95) | 117.86  (107.51,127.14) | 63.09  (57.9,68.47) | 93.32  (84.36,102.14) | 50.96  (45.57,57.17) | 49.31  (43.87,55.56) |
| Percent change of rate 1990–2019,% | 10.92  (3.88,16.94) | | -30.49  (-34.58,-26.67) | | 14.27  (6.92,21.07) | | 75.77  (59.70,91.26) | | 47.91  (31.09,64.55) | | -3.23  (-16.51,9.58) | |
| **Age-standardized mortality rate** | | | | | | | | | | | | |
| Rate per 100,000 | 170.45  (159.61,176.94) | 117.95  (107.83,125.92) | 162.39  (150.62,168.15) | 67.1  (60.07,71.54) | 209.2  (196.25,216.23) | 135.41  (122.68,144.44) | 143.11  (133.18,152.13) | 134.12  (121.51,145.22) | 144.21  (132.05,156.58) | 136.59  (122.96,149.50) | 139.2  (124,156.79) | 127.99  (113.13,143.90) |
| Percent change of rate 1990–2019,% | -30.8  (-34.83,-27.17) | | -58.68  (-60.30,-56.69) | | -35.28  (-39.01,-31.69) | | -6.28  (-14.41,1.84) | | -5.28  (-15.25,5.09) | | -8.05  (-20.59,3.17) | |
| **APC model estimates** | | | | | | | | | | | | |
| Net drift of mortality y ,% per year | -1.1  (-1.17,-1.04) | | -2.84  (-3.05,-2.64) | | -1.99  (-2.18,-1.80) | | -0.35  (-0.42,-0.28) | | -0.26  (-0.38,-0.15) | | -0.47  (-0.54,-0.39) | |

**Notes:** All-age mortality=crude mortality rate. The age-standardized mortality rate is computed by direct standardization with the global standard population in GBD 2019.

* Parentheses for all GBD health estimates indicate 95% uncertainty intervals; parentheses for net drift indicate 95% confidence intervals; Net drifts are estimates derived from the age-period-cohort model and denote the overall annual percentage change in mortality, which captures the contribution of the effects from calendar time and successive birth cohorts; IHD = Ischemic heart disease; SDI = Socio-demographic Index; APC = age-period-cohort

**Supplementary Table 2. The APC analysis results of all 204 countries and regions.**

|  | **Deaths** | | **All-age mortality** | | **Age-standardized mortality** | | **Net drift of mortality from APC model,% per year** |
| --- | --- | --- | --- | --- | --- | --- | --- |
|  | **Number in 2019** | **Percent change 1990-2019, %** | **Rate in 2019** | **Percent change 1990-2019, %** | **Rate in 2019** | **Percent change 1990-2019, %** |  |
| Afghanistan | 34627.79(26988.79,42664.91) | 32.62(0.41,68.89) | 90.47(70.51,111.46) | -60.44(-70.05,-49.62) | 320.87(253.97,385.29) | -21.75(-39.03,-4.26) | -1.38(-1.46,-1.29) |
| Albania | 6008.15(4725.56,7562.18) | 112.37(66.27,168.26) | 220.86(173.71,277.99) | 158.39(102.31,226.4) | 145.82(115.45,183.28) | -10.58(-29.92,12.60) | -0.12(-0.44,0.20) |
| Algeria | 58692.07(47672.64,71562.90) | 83.07(43.09,131.01) | 140.25(113.92,171.01) | 10.61(-13.55,39.57) | 237.25(197.13,282.69) | -40.01(-51.65,-26.49) | -2.64(-3,-2.29) |
| American Samoa | 64.48(55.43,74.37) | 118.33(82.84,159.71) | 116.16(99.87,133.99) | 90.51(59.54,126.61) | 151.20(131.24,173.21) | -0.84(-15.75,17.73) | 0.10(-2.35,2.62) |
| Andorra | 75.76(59.19,94.24) | 125.7(56.72,203.18) | 91.20(71.26,113.45) | 46.98(2.06,97.43) | 47.77(37.09,59.62) | -41.17(-57.86,-22.59) | -2.37(-7.6,3.15) |
| Angola | 9300.47(7170.48,11820.73) | 144.86(78.51,239.31) | 30.86(23.79,39.22) | -16.17(-38.89,16.17) | 112.72(86.2,140.65) | -8.11(-32.59,23.28) | -0.87(-1.05,-0.69) |
| Antigua and Barbuda | 78.19(68.19,87.94) | 4.78(-8.72,17.58) | 88.36(77.06,99.38) | -28.10(-37.36,-19.32) | 89.27(78.21,99.86) | -32.88(-41.19,-25.07) | -2.57(-5.63,0.59) |
| Argentina | 45371.19(41257.06,48541.68) | -8.48(-13.69,-2.83) | 100.57(91.45,107.59) | -32.81(-36.64,-28.66) | 82.07(74.77,87.71) | -51.87(-54.39,-49.08) | -2.82(-3.05,-2.6) |
| Armenia | 9255.39(7880.28,10687.61) | 31.63(13.71,50.56) | 306.50(260.96,353.93) | 48.85(28.58,70.25) | 237.45(201.67,272.91) | -27.80(-37.22,-17.97) | -1.99(-2.36,-1.62) |
| Australia | 26633.26(22749.38,28854.96) | -19.52(-26.16,-14.79) | 108.41(92.6,117.45) | -44.77(-49.32,-41.52) | 56.09(48.62,60.44) | -68.35(-70.41,-66.72) | -4.43(-4.81,-4.05) |
| Austria | 18087.02(15759.16,19911.39) | -10.97(-18.68,-5.58) | 202.86(176.75,223.32) | -22.43(-29.15,-17.73) | 83.31(73.55,90.96) | -50.10(-53.82,-47.4) | -4.07(-4.64,-3.49) |
| Azerbaijan | 27208.68(23829.09,31097.18) | 81.62(57.56,110.25) | 264.71(231.83,302.54) | 29.53(12.38,49.96) | 452.58(399.78,506.77) | 35.76(19.27,54.74) | -1.24(-1.56,-0.92) |
| Bahamas | 326.27(271.96,397.62) | 69.17(39.55,104.19) | 86.56(72.15,105.49) | 15.10(-5.05,38.93) | 92.4(76.87,111.92) | -34.51(-45.38,-21.26) | -1.56(-2.71,-0.41) |
| Bahrain | 863.35(697.71,1059.73) | 53.98(20.93,99.34) | 59.84(48.36,73.46) | -45.78(-57.42,-29.8) | 155.09(129.29,186.67) | -64.95(-71.4,-55.90) | -5.30(-5.83,-4.78) |
| Bangladesh | 131010.17(102869.37,160333.9) | 175.72(114.66,253.89) | 82.26(64.59,100.67) | 88.8(46.99,142.33) | 111.23(86.84,135.23) | -2.67(-22.99,23.13) | 0.05(-0.22,0.33) |
| Barbados | 342.34(291.25,394.51) | -10.35(-23.33,2.37) | 114.97(97.81,132.49) | -23.56(-34.63,-12.71) | 69.84(59.53,80.56) | -45.13(-53.08,-37.52) | -2.85(-4.68,-0.98) |
| Belarus | 54617.86(44896.82,66662.76) | 35.95(12.24,65.17) | 574.88(472.56,701.66) | 49.83(23.69,82.03) | 334.16(274.57,408.41) | 0.55(-17.19,22.52) | -1.00(-1.76,-0.22) |
| Belgium | 15145.69(13337.06,16362.03) | -32.05(-36.67,-27.94) | 132.63(116.8,143.29) | -40.62(-44.65,-37.02) | 54.80(48.83,58.85) | -61.86(-63.85,-59.84) | -4.17(-4.55,-3.79) |
| Belize | 210.07(184.29,237.32) | 67.95(47.06,91.72) | 51.23(44.94,57.87) | -23.87(-33.34,-13.1) | 84.86(73.99,95.55) | -39.85(-46.92,-31.58) | -2.18(-3.52,-0.82) |
| Benin | 4432.09(3575.43,5561.05) | 103.66(65.2,157.75) | 34.99(28.23,43.91) | -21.97(-36.71,-1.25) | 113.10(92.68,137.52) | -9.86(-24.99,11.70) | -0.39(-0.69,-0.09) |
| Bermuda | 109.67(91.48,129.7) | -20.66(-31.79,-7.07) | 171.27(142.87,202.57) | -26.33(-36.66,-13.71) | 78.13(65.46,92.38) | -67.94(-72.25,-62.45) | -4.5(-8.5,-0.33) |
| Bhutan | 663.09(517.21,810.63) | 165.57(94.15,265.59) | 87.91(68.57,107.48) | 115.54(57.58,196.72) | 134.54(105.85,162.82) | 10.87(-17.64,48.37) | -0.37(-1.04,0.31) |
| Bolivia (Plurinational State of) | 7714.39(5470.41,10112.19) | 110.86(63.52,171.9) | 64.22(45.54,84.19) | 12.72(-12.59,45.35) | 105.91(76.21,136.33) | -24.58(-40.65,-3.46) | -1.76(-1.99,-1.53) |
| Bosnia and Herzegovina | 8817.46(7236.77,10659.7) | 20.82(-0.04,45.50) | 267.20(219.3,323.02) | 66.14(37.46,100.08) | 162.94(133.68,195.74) | -30.83(-42.3,-17.43) | -2.76(-3.11,-2.40) |
| Botswana | 1408.85(1038.76,1875.25) | 152.18(83.24,242.85) | 60.24(44.42,80.18) | 40.36(1.99,90.82) | 130.96(98.83,170.58) | 6.86(-19.13,42.84) | -0.57(-1.08,-0.05) |
| Brazil | 171246.34(156180.04,180511.23) | 46.06(37.65,53.13) | 79.04(72.08,83.31) | 0.33(-5.44,5.19) | 74.90(67.93,79.12) | -52.55(-54.9,-50.31) | -2.14(-2.22,-2.07) |
| Brunei Darussalam | 261.84(233.76,294.38) | 75.54(50.88,105.33) | 59.90(53.48,67.35) | 3.83(-10.76,21.45) | 132.31(117.58,147.23) | -35.15(-43.42,-25.22) | -1.89(-2.77,-1.00) |
| Bulgaria | 34450.31(28594.84,40548.68) | -3.75(-19.79,14.65) | 496.79(412.35,584.73) | 20.49(0.41,43.52) | 239.07(197.94,281.15) | -37.09(-47.4,-25.13) | -2.35(-2.74,-1.97) |
| Burkina Faso | 9474.40(7865.22,11195.78) | 153.79(105.73,214.03) | 41.75(34.66,49.34) | 6.90(-13.35,32.27) | 130.19(110.76,152.07) | 19.63(-0.93,46.13) | 0.74(0.51,0.97) |
| Burundi | 4028.67(3025.9,5299.93) | 52.5(9.11,114.02) | 33.76(25.35,44.41) | -28.81(-49.07,-0.10) | 113.58(86.32,147.64) | -14.5(-37.79,16.34) | -1.01(-1.25,-0.76) |
| Cabo Verde | 568.88(484.28,635.3) | 85.82(60.56,113.51) | 100.94(85.93,112.73) | 15.93(0.17,33.21) | 138.83(118.43,155.21) | 8.52(-6.03,24.31) | -0.8(-1.94,0.35) |
| Cambodia | 11456.16(9345.91,13756.9) | 147.54(87.78,214.1) | 69(56.29,82.86) | 54.69(17.35,96.28) | 118.57(96.93,139.99) | -3.41(-26.25,19.68) | -0.91(-1.08,-0.73) |
| Cameroon | 10618.86(8305.74,13690.64) | 199.34(121.48,310.18) | 36.49(28.54,47.04) | 6.88(-20.92,46.46) | 115.52(93.18,145.7) | 8.66(-17.62,44.19) | 0.64(0.44,0.84) |
| Canada | 48774.05(42888.85,52708.67) | -4.97(-10.61,0.84) | 133.55(117.44,144.33) | -29.07(-33.29,-24.74) | 63.95(56.89,68.7) | -60.64(-62.43,-58.54) | -3.44(-3.74,-3.13) |
| Central African Republic | 2524.77(1888.47,3318.40) | 73.38(31.01,129.64) | 47.64(35.63,62.61) | -10.23(-32.17,18.90) | 146.61(109.7,194.31) | -3.33(-25.26,25.09) | -0.04(-0.36,0.28) |
| Chad | 5367.71(4388.84,6613.18) | 95.07(56.82,144.52) | 32.73(26.76,40.33) | -28.37(-42.41,-10.21) | 120.14(99.28,145.38) | 4.75(-13.72,29.48) | 0.32(0.05,0.6) |
| Chile | 11986.39(10801.63,12844.34) | 8.91(1.87,15.53) | 65.87(59.35,70.58) | -20.52(-25.66,-15.69) | 50.38(45.32,54.01) | -62.52(-64.67,-60.34) | -1.95(-2.38,-1.52) |
| China | 1874006.90(1612111.34,2131821.5) | 208.35(159.08,267.32) | 131.75(113.34,149.88) | 156.61(115.61,205.68) | 116.41(100.29,131.52) | 17.61(0.22,38.94) | 0.02(-0.17,0.21) |
| Colombia | 41709.13(32538.63,51907.21) | 84.95(47.21,128.61) | 87.30(68.11,108.65) | 26(0.29,55.74) | 75.34(59.01,93.92) | -50.1(-60.13,-38.27) | -2.86(-2.97,-2.75) |
| Comoros | 443.16(335.96,579.75) | 113.12(55.31,225.73) | 62.04(47.03,81.16) | 38.95(1.26,112.37) | 105.59(80.15,137.20) | -6.8(-29.91,33.15) | -0.35(-1.29,0.60) |
| Congo | 2796.80(2094.43,3720.67) | 88.49(42.83,143.91) | 53.11(39.77,70.66) | -12.49(-33.69,13.24) | 140.89(105.16,183.06) | -17.56(-35.3,4.07) | -1.53(-1.87,-1.20) |
| Cook Islands | 24.31(20.51,28.75) | 53.32(20.96,94.13) | 135.18(114.05,159.82) | 62.14(27.92,105.29) | 103.41(87.32,122.36) | -25.46(-40.29,-6.08) | -0.94(-5.59,3.94) |
| Costa Rica | 3708.17(2908.44,4605.9) | 75.94(43.27,117.27) | 78.62(61.66,97.65) | 13.39(-7.67,40.03) | 71.47(55.99,88.87) | -45.53(-55.71,-32.56) | -2.07(-2.48,-1.66) |
| Côte d'Ivoire | 9649.60(7568.93,12073.99) | 134.46(85.25,196.14) | 36.87(28.92,46.13) | 9.53(-13.46,38.35) | 121.97(99.42,146.76) | -16.44(-30.91,1.28) | -0.85(-1.02,-0.69) |
| Croatia | 13197.44(10663.60,15821.62) | -11.17(-27.08,6.91) | 310.68(251.03,372.46) | 2.47(-15.88,23.33) | 143.78(116.19,172.7) | -47.53(-56.93,-37.16) | -3.24(-4.2,-2.28) |
| Cuba | 22271.99(18669.60,26462) | 11.63(-4.68,32.07) | 196.08(164.37,232.97) | 6.46(-9.1,25.96) | 109.85(92.08,130.99) | -46.64(-54.8,-36.8) | -2.88(-3.15,-2.61) |
| Cyprus | 1595.73(1393.87,1894.86) | 15.42(-3.95,36.64) | 121.49(106.12,144.26) | -31.63(-43.11,-19.07) | 95.5(82.8,114) | -58.15(-65.04,-50.65) | -3.47(-4.34,-2.6) |
| Czechia | 32959.58(27314.47,38619.92) | -22.99(-34.94,-10.7) | 309.67(256.63,362.85) | -25.49(-37.06,-13.6) | 148.97(123.54,174.69) | -54.49(-61.49,-47.08) | -4.44(-4.88,-3.99) |
| Democratic People's Republic of Korea | 35798.22(30115.83,42400.58) | 168.98(113.14,244.21) | 136.46(114.8,161.63) | 115.89(71.07,176.27) | 126.54(106.78,150.01) | 17.51(-6.46,49.68) | 0.64(0.53,0.74) |
| Democratic Republic of the Congo | 31794.54(23189.24,42848.74) | 106.76(58.63,169.05) | 36.27(26.45,48.87) | -8.99(-30.17,18.43) | 114.72(83.9,153.66) | -10.25(-29.48,13.51) | -0.57(-0.67,-0.47) |
| Denmark | 7169.75(6278.23,7763.03) | -59.92(-62.73,-57.23) | 123.56(108.19,133.78) | -64.47(-66.96,-62.08) | 55.55(49.11,59.99) | -73.51(-75.18,-71.75) | -6.11(-7.03,-5.18) |
| Djibouti | 495.70(346.13,686.14) | 415.54(251.4,621.28) | 41.21(28.78,57.05) | 108.31(41.99,191.44) | 115.99(84.39,154.53) | 16.07(-16.39,54.26) | 0.52(-0.4,1.45) |
| Dominica | 81.17(68.56,96.16) | -24.94(-36.79,-11.32) | 118.18(99.82,140) | -19.12(-31.89,-4.44) | 89.38(75.54,105.94) | -39.10(-48.72,-27.91) | -2.15(-5.42,1.23) |
| Dominican Republic | 15306.15(12105.33,19101.6) | 248.99(170.48,341.18) | 140.66(111.24,175.54) | 131.01(79.04,192.03) | 175.57(139.73,217.55) | 25.89(-0.99,57.93) | 1.51(1.3,1.73) |
| Ecuador | 10683.99(8640.53,13328.87) | 126.88(85.03,181.34) | 60.74(49.13,75.78) | 29.33(5.47,60.37) | 81.81(66.65,101.43) | -21.31(-35.19,-3.05) | -1.08(-1.34,-0.83) |
| Egypt | 181884.57(138958.56,233632.32) | 88.00(43.65,138.61) | 183.59(140.26,235.83) | 5.69(-19.24,34.15) | 359.27(281.82,447.03) | -12.54(-32.42,8.77) | -0.50(-0.65,-0.36) |
| El Salvador | 6481.97(5054.83,8121.22) | 84.45(45.29,129.30) | 103.61(80.8,129.81) | 55.30(22.33,93.06) | 100.37(78.13,125.94) | -18.69(-36.45,2.47) | -1.64(-1.88,-1.40) |
| Equatorial Guinea | 349.18(248.78,488.33) | 47.95(-1.27,117.32) | 24.59(17.52,34.39) | -55.16(-70.08,-34.13) | 97.07(69.42,129.45) | -31.44(-54.59,-0.99) | -3.03(-3.93,-2.13) |
| Eritrea | 2272.55(1745.48,2938.5) | 210.07(121.55,343.40) | 33.86(26.01,43.79) | 38.67(-0.92,98.30) | 113.46(89.36,142.45) | 20.05(-14.16,64.86) | 0.32(-0.03,0.67) |
| Estonia | 4434.19(3512.33,5892.61) | -36.51(-48.87,-15.05) | 337.88(267.63,449.01) | -24.11(-38.88,1.55) | 144.16(114.15,191.36) | -60.74(-68.56,-47.91) | -5.13(-6.17,-4.08) |
| Eswatini | 548.82(405.25,734.3) | 106.83(50.25,182.74) | 48.05(35.48,64.29) | 46.09(6.12,99.7) | 121.58(91.33,158.57) | 7.49(-21.07,42.36) | 0.83(0.03,1.63) |
| Ethiopia | 28409.13(21139.41,35645.21) | 51.30(-9.95,118.46) | 26.4(19.65,33.13) | -27.73(-56.99,4.35) | 84.24(62.56,105.75) | -23.60(-54.12,8.08) | -2.47(-2.58,-2.36) |
| Fiji | 1574.64(1255.81,1939.12) | 69.04(29.26,124.63) | 172.80(137.81,212.80) | 40.86(7.71,87.18) | 249.94(205.02,302.15) | -13.91(-32.42,10.31) | -0.84(-1.27,-0.41) |
| Finland | 14812.33(12840.46,16035.81) | -7.55(-15.29,-0.45) | 267.66(232.02,289.76) | -16.3(-23.31,-9.88) | 100.57(88.89,108.53) | -55.5(-58.56,-52.17) | -4.20(-4.89,-3.50) |
| France | 68324.88(57931.92,75789.99) | -10.63(-18.08,-3.98) | 103.2(87.5,114.48) | -22.02(-28.52,-16.21) | 38.41(33.46,41.9) | -56.13(-58.77,-53.6) | -3.17(-3.55,-2.79) |
| Gabon | 986.71(770.73,1222.05) | 57.83(23.42,97.02) | 56.38(44.04,69.83) | -10.58(-30.07,11.63) | 117.37(91.71,146.47) | -11.07(-28.47,9.75) | -0.93(-1.53,-0.32) |
| Gambia | 1205.67(977.63,1457.94) | 241.14(154.02,343.85) | 53.68(43.53,64.92) | 50.68(12.19,96.04) | 151.23(125.37,180.37) | 13.45(-12.13,42.75) | 0.55(-0.1,1.21) |
| Georgia | 13916.83(11825.92,16058.63) | -36.71(-45.97,-24.46) | 379.75(322.69,438.19) | -4.87(-18.78,13.55) | 212.6(181.64,245.68) | -50.53(-57.55,-41.15) | -3.43(-3.92,-2.95) |
| Germany | 184322.65(164517.17,197583.29) | -32.11(-36.04,-27.03) | 217.07(193.75,232.69) | -36.08(-39.79,-31.31) | 81.42(73.29,86.75) | -60.92(-62.92,-58.14) | -4.26(-4.77,-3.75) |
| Ghana | 15888.28(13341.18,18985.48) | 170.33(112.68,239.04) | 50.38(42.3,60.20) | 28.72(1.27,61.44) | 128.55(110.06,151.39) | 3.02(-16.55,26.71) | -0.13(-0.28,0.03) |
| Greece | 26142.45(22942.03,28068.32) | 26.47(17.21,34.62) | 252.9(221.94,271.53) | 27.11(17.8,35.31) | 91.92(82.51,97.66) | -35.87(-39.09,-32.44) | -1.4(-1.72,-1.09) |
| Greenland | 56.07(46.55,65.89) | 3.15(-17.59,24.84) | 99.79(82.84,117.26) | 2.03(-18.49,23.48) | 96.19(81.2,111.86) | -52.63(-61.16,-43.72) | -3.51(-8.65,1.92) |
| Grenada | 105.20(96.08,114.18) | -30.35(-36.63,-23.31) | 101.92(93.08,110.62) | -42.19(-47.40,-36.34) | 107.05(97.17,116.31) | -44.05(-49.19,-38.74) | -2.40(-4.84,0.1) |
| Guam | 320.39(273.41,374.41) | 186.22(136.36,244.61) | 187.77(160.24,219.43) | 129.41(89.44,176.21) | 171.76(146.81,200.85) | -14.32(-29.31,2.29) | 0.35(-0.88,1.60) |
| Guatemala | 9408.46(7651.45,11328.46) | 107.27(67.71,156.74) | 52.93(43.04,63.73) | -7.11(-24.84,15.06) | 106.32(87.43,126.24) | -35.37(-46.47,-22.26) | -2.69(-2.85,-2.53) |
| Guinea | 5826.11(4758.28,7226.93) | 93.59(51.24,149.02) | 46.08(37.64,57.16) | -5.28(-26,21.85) | 123.67(101.51,152.11) | 13.54(-10.17,43.13) | 1.17(0.9,1.45) |
| Guinea-Bissau | 936.37(735.13,1173.43) | 70.19(23.2,138.71) | 49.25(38.67,61.72) | -9.83(-34.72,26.48) | 164.54(131.86,202.57) | -1.09(-26.15,34.31) | 0.16(-0.37,0.69) |
| Guyana | 1047.07(836.53,1293.57) | 14.81(-10.55,46.1) | 135.86(108.54,167.84) | 14.71(-10.63,45.98) | 192.95(155.81,233.9) | -29.66(-44.01,-12.64) | -1.42(-1.98,-0.85) |
| Haiti | 11148.69(7989.87,15413.41) | 67.68(26.85,120.71) | 89.89(64.42,124.28) | -14.07(-34.99,13.11) | 196.02(142.67,265.82) | -19.90(-37.45,2.89) | -0.85(-1.03,-0.68) |
| Honduras | 7606.49(6221.77,9374.46) | 247.39(188.84,327.79) | 77.50(63.39,95.52) | 66.66(38.57,105.24) | 154.84(126.87,189.4) | 27.61(7.1,56.65) | -0.74(-1.03,-0.44) |
| Hungary | 35930.56(30178.29,41876.6) | -5.67(-19.94,10.17) | 371.4(311.94,432.86) | 1.34(-14,18.35) | 174.62(146.49,203.95) | -38.86(-47.65,-28.68) | -3.38(-3.73,-3.03) |
| Iceland | 436.30(368.14,482.75) | -14.72(-23.24,-7.02) | 126.51(106.75,139.98) | -37.2(-43.48,-31.53) | 66.24(56.82,72.98) | -61.21(-64.78,-57.65) | -3.96(-5.72,-2.16) |
| India | 1519123.85(1311371.82,1745628.96) | 144.32(100.55,185.66) | 109.23(94.3,125.52) | 50.31(23.38,75.74) | 150.51(129.37,172.58) | -11.74(-26.57,2.47) | -0.55(-0.76,-0.35) |
| Indonesia | 245343.96(207414.43,275665.76) | 150.71(104.72,198.88) | 94.56(79.94,106.24) | 79.12(46.26,113.54) | 140.33(119.78,154.99) | 18.87(-1.51,41.46) | 0.37(0.31,0.43) |
| Iran (Islamic Republic of) | 102798.78(94454.77,111215.26) | 84.66(65.27,100.03) | 121.95(112.05,131.93) | 28.24(14.77,38.91) | 163.56(148.98,176.17) | -42.75(-47.92,-38.07) | -2.90(-3.02,-2.79) |
| Iraq | 46847.94(38262.60,55510.86) | 117.33(71.65,167.11) | 111.23(90.84,131.79) | -9.20(-28.29,11.59) | 255.4(214.11,292.61) | -17.93(-33.55,-2.03) | -2.01(-2.13,-1.88) |
| Ireland | 5809.69(5084.05,6282.99) | -36.16(-42.08,-31.84) | 118.32(103.54,127.95) | -53.18(-57.52,-50.01) | 74.7(65.6,80.72) | -67.7(-70.39,-65.68) | -5.17(-5.87,-4.45) |
| Israel | 6165.95(5376.37,6664.33) | -24.18(-30.75,-19.33) | 66.23(57.75,71.59) | -59.59(-63.09,-57.01) | 48.13(42.38,51.86) | -73.13(-75.08,-71.63) | -6.09(-6.54,-5.63) |
| Italy | 101158.72(85415.46,111561.45) | -2.37(-12.92,3.10) | 167.72(141.62,184.97) | -8.06(-17.99,-2.91) | 55.27(47.69,60.39) | -54.11(-57.59,-52.14) | -3.46(-3.79,-3.13) |
| Jamaica | 2109.36(1737.58,2520.37) | 38.92(14.98,66.85) | 75.05(61.82,89.67) | 16.82(-3.31,40.31) | 64.28(52.47,77.27) | -23.37(-37.08,-7.30) | -0.55(-1.19,0.09) |
| Japan | 140588.25(111885.77,156599.56) | 22.19(5.53,31.76) | 110.02(87.56,122.55) | 20.36(3.94,29.78) | 29.87(25.15,32.50) | -60.78(-63.80,-58.87) | -2.37(-2.77,-1.96) |
| Jordan | 6110.96(5195.84,7285.93) | 158.16(111.47,221.95) | 52.51(44.65,62.61) | -16.29(-31.43,4.39) | 121.92(103.15,144.05) | -45.91(-55.02,-34.59) | -3.08(-3.33,-2.83) |
| Kazakhstan | 33047.17(28771.2,37362.48) | 0.98(-11.2,13.68) | 179.68(156.43,203.14) | -10.14(-20.97,1.17) | 251.41(219.73,281.88) | -17.09(-26.29,-7.71) | -3.20(-3.72,-2.68) |
| Kenya | 13716.65(10677.84,17133.31) | 228.87(180.57,283.66) | 27.31(21.26,34.11) | 51.86(29.56,77.16) | 82.59(63.74,103.66) | 23.65(6.18,42.46) | 1.37(1.15,1.58) |
| Kiribati | 157.61(125.4,196.28) | 64.92(27.12,116.24) | 132.86(105.71,165.47) | 2.97(-20.63,35.01) | 249.8(202.36,300.91) | -5.79(-24.76,19.79) | -0.44(-1.59,0.73) |
| Kuwait | 2599.19(2165.34,3105.42) | 154.7(113.73,204.20) | 58.72(48.92,70.15) | 1.24(-15.05,20.91) | 108.53(90.73,129.20) | -44.22(-52.68,-34.04) | -1.73(-2.05,-1.40) |
| Kyrgyzstan | 11482.94(10201.55,12763.47) | 71.76(53.32,90.83) | 175.7(156.1,195.3) | 17.27(4.69,30.29) | 320.39(283.02,354.78) | 36.12(22.12,50.21) | -0.73(-1.23,-0.22) |
| Lao People's Democratic Republic | 5674.81(4637.44,6808.97) | 92.19(43.75,149.33) | 79.28(64.78,95.12) | 11.47(-16.63,44.61) | 162.08(134.69,189.79) | -5.52(-26.93,19.87) | -0.60(-0.82,-0.39) |
| Latvia | 9020.75(7678.49,10553.85) | -21.19(-31.79,-8.50) | 470.99(400.9,551.03) | 9.40(-5.31,27.01) | 200.43(171.64,234.57) | -39.85(-47.71,-30.17) | -3.46(-4.64,-2.28) |
| Lebanon | 12251.42(8865.73,14092.29) | 81.06(35.06,111.96) | 236.65(171.25,272.21) | 14.54(-14.56,34.09) | 241.23(174.11,277.12) | -31.59(-48.89,-20.45) | -1.21(-1.4,-1.02) |
| Lesotho | 1270.44(934.23,1652.86) | 82.22(35.53,145.94) | 60.74(44.67,79.02) | 57.45(17.11,112.50) | 123.63(91.41,157.92) | 46.06(10.09,92.30) | 2.63(2.02,3.25) |
| Liberia | 1888.23(1473.16,2448.13) | 51.82(15.82,99.05) | 39.42(30.76,51.11) | -37.74(-52.51,-18.37) | 117.44(93.65,148.15) | -14.04(-33.47,10.41) | -0.39(-0.83,0.05) |
| Libya | 7826.81(6177.05,10362.77) | 144.29(90.31,215.04) | 116.2(91.71,153.85) | 53.67(19.71,98.17) | 171.3(135.45,226.05) | -9.78(-29.12,14.75) | -0.46(-0.67,-0.25) |
| Lithuania | 14637.81(12214.35,17199.99) | -4.95(-18.52,10.60) | 523.86(437.13,615.56) | 24.96(7.13,45.41) | 222.93(186.66,262.51) | -36.70(-45.96,-25.98) | -2.36(-3.4,-1.30) |
| Luxembourg | 616.55(517.73,702.17) | -28.36(-37.70,-19.34) | 99.68(83.70,113.52) | -55.84(-61.60,-50.29) | 54.17(45.97,61.62) | -66.74(-70.82,-62.62) | -4.96(-6.55,-3.35) |
| Madagascar | 10713.99(7666.17,14408.63) | 100.97(47.96,167.53) | 40.14(28.72,53.98) | -10.01(-33.75,19.80) | 132.35(98.19,172.78) | 2.87(-22.65,33.79) | -0.20(-0.35,-0.06) |
| Malawi | 5470.69(4285.82,6783.42) | 79.53(35.54,135.03) | 29.66(23.24,36.78) | -6.98(-29.77,21.78) | 90.11(71.03,111.30) | -7.34(-29.13,18.19) | -0.67(-0.88,-0.47) |
| Malaysia | 34010.47(27126.41,41375.43) | 158.15(104.13,217.73) | 108.65(86.66,132.18) | 45.6(15.14,79.21) | 145.71(117.74,175.96) | -12.00(-28.98,6.88) | -0.13(-0.43,0.16) |
| Maldives | 307.84(259.64,359.59) | 66.39(34.54,103.78) | 61.76(52.09,72.15) | -25.91(-40.09,-9.27) | 120.91(101.79,141.05) | -54.43(-62.96,-44.77) | -4.21(-5.21,-3.20) |
| Mali | 7595.85(6053.46,9385.72) | 96.63(54.49,145.72) | 34.66(27.62,42.82) | -22.19(-38.87,-2.77) | 116.05(93.53,140.92) | -9.15(-28.53,10.97) | -0.47(-0.71,-0.24) |
| Malta | 919.97(794.81,1017.21) | 8.22(-3.17,19.18) | 209.45(180.96,231.59) | -8.67(-18.28,0.59) | 90.83(78.79,100.58) | -57.58(-61.64,-53.3) | -3.14(-4.47,-1.79) |
| Marshall Islands | 80.32(60.21,106.99) | 111.80(67.43,171.08) | 141.30(105.93,188.22) | 70.39(34.69,118.08) | 277.78(214.38,356.28) | 5.56(-15.27,30.73) | 0.33(-1.44,2.14) |
| Mauritania | 1826.64(1485.55,2256.08) | 43.44(12.44,78.64) | 45.50(37.01,56.2) | -26.16(-42.12,-8.04) | 106.02(87.31,127.8) | -30.42(-44.31,-14.21) | -1.68(-2.17,-1.18) |
| Mauritius | 1663.44(1381.65,1977.24) | -3.52(-19.33,14.44) | 130.30(108.22,154.88) | -16.87(-30.49,-1.39) | 103.82(86.23,123.12) | -60.74(-66.98,-53.57) | -4.23(-4.71,-3.75) |
| Mexico | 106311.06(91979.69,120365.05) | 167.93(134.78,202.77) | 85.09(73.62,96.34) | 83.33(60.65,107.16) | 100(85.98,112.92) | -12.6(-23.12,-1.77) | -0.04(-0.18,0.09) |
| Micronesia (Federated States of) | 173.2(123.34,227.23) | 56.44(8.5,113.53) | 169.61(120.79,222.52) | 59.76(10.8,118.06) | 287.23(216.68,365.42) | 7.62(-20,41.86) | 0.15(-1.02,1.33) |
| Monaco | 79.69(64.13,92.15) | -22.78(-37.45,-4.13) | 212.09(170.69,245.27) | -37.44(-49.32,-22.33) | 67.21(53.8,77.91) | -47.46(-58.26,-34.05) | -2.5(-8.69,4.11) |
| Mongolia | 4905.53(3934.3,6127.89) | 41.79(11.35,84.98) | 144.81(116.14,180.89) | -9.86(-29.21,17.60) | 311.63(260.94,376.5) | -23.1(-36.67,-4.23) | -2.06(-2.34,-1.78) |
| Montenegro | 1488.24(1252.55,1725.66) | 62.25(35.54,92.75) | 239.91(201.91,278.18) | 63.66(36.72,94.43) | 164.06(138.59,189.24) | 0.99(-14.87,18.82) | -1.17(-2.22,-0.11) |
| Morocco | 72011.52(56906.33,84500.53) | 104.02(63.76,138.82) | 200.3(158.28,235.04) | 43.55(15.23,68.04) | 278.54(224.66,321.41) | -9.04(-25.23,4.81) | -1.20(-1.40,-0.99) |
| Mozambique | 8936.37(6993.76,11427.77) | 137.84(79.44,217.92) | 30.26(23.69,38.70) | 5.28(-20.57,40.73) | 102.04(80.3,129.34) | 26.03(-5.4,63.45) | 1.78(1.58,1.99) |
| Myanmar | 40583.20(35581.09,46986.38) | 40.00(8.12,82.69) | 74.22(65.08,85.93) | 5.25(-18.72,37.34) | 104.10(91.37,119.61) | -28.63(-43.12,-9.36) | -2.22(-2.31,-2.13) |
| Namibia | 1373.77(1097.81,1670.37) | 87.21(44.17,142.38) | 57.17(45.68,69.51) | 9.82(-15.43,42.18) | 115.08(93.03,138.54) | -6.35(-27.06,18.64) | -0.81(-1.45,-0.17) |
| Nauru | 12.98(10.37,16.08) | 13.41(-8.31,40.98) | 122.98(98.29,152.44) | 10.22(-10.89,37.01) | 356.23(294.34,424.75) | 6.79(-11.51,27.78) | 0.22(-2.98,3.53) |
| Nepal | 23742.58(18714.36,28810.98) | 165.1(99.94,239.34) | 78.06(61.53,94.72) | 70.28(28.42,117.96) | 123.99(98.62,148.27) | 13.36(-13.74,43.35) | -0.15(-0.26,-0.03) |
| Netherlands | 17642.13(15551.69,19086.38) | -40.22(-44.03,-36.66) | 102.83(90.64,111.25) | -48.00(-51.32,-44.91) | 47.40(42.06,51.13) | -67.54(-69.47,-65.74) | -5.37(-5.97,-4.78) |
| New Zealand | 6491.93(5671.95,6994.93) | -9.55(-16.46,-4.44) | 144.4(126.16,155.59) | -31.25(-36.5,-27.36) | 75.00(66.21,80.44) | -59.77(-62.19,-57.71) | -4.37(-4.99,-3.74) |
| Nicaragua | 5091.06(4368.91,5799.64) | 264.77(214.25,315.34) | 78.2(67.11,89.08) | 117.79(87.63,147.98) | 148.33(126.91,166.41) | 38.76(20.92,56.23) | 0.10(-0.25,0.44) |
| Niger | 6756.26(5241.53,8649.07) | 155.51(99.53,233.22) | 29.00(22.50,37.13) | -12.00(-31.28,14.76) | 118.13(93.96,146.38) | -9.46(-27.71,13.77) | -0.46(-0.71,-0.22) |
| Nigeria | 69581.50(50603.23,89624.79) | 55.76(-1.77,110.74) | 32.39(23.56,41.72) | -34.61(-58.76,-11.53) | 105.21(76.6,132.53) | -17.73(-48.96,8.89) | -1.19(-1.28,-1.10) |
| Niue | 4.42(3.56,5.22) | -12.61(-29.11,6.70) | 264.47(212.98,312.12) | 21.64(-1.34,48.52) | 208.81(167.88,247.91) | -5.77(-23.81,15.91) | -0.69(-10.38,10.05) |
| North Macedonia | 5155.80(4224.2,6227.61) | 42.00(15.82,72.26) | 239.50(196.23,289.29) | 32.94(8.43,61.27) | 209.93(176.09,247.29) | -8.83(-23.64,9.07) | -2.03(-2.45,-1.62) |
| Northern Mariana Islands | 64.45(55.14,73.98) | 243.61(171.36,328.15) | 151.67(129.76,174.10) | 267.09(189.9,357.4) | 148.79(128.85,170.16) | 19.44(-1.93,44.49) | 0.75(-2.32,3.92) |
| Norway | 6270.68(5449.27,6831.47) | -51.92(-55.65,-48.26) | 117.23(101.88,127.72) | -61.83(-64.79,-58.92) | 55.47(48.96,59.95) | -69.02(-71.04,-66.69) | -5.68(-6.4,-4.96) |
| Oman | 3411.65(3045.72,3828.43) | 44.28(16.12,85.11) | 74.43(66.44,83.52) | -38.84(-50.78,-21.53) | 329.85(296.03,364.09) | -29.84(-41.94,-13.02) | -2.76(-3.3,-2.21) |
| Pakistan | 183409.43(152232.54,220359.19) | 141.35(95.89,197.3) | 81.86(67.94,98.35) | 21.54(-1.35,49.72) | 189.25(158.03,225.54) | 29.62(7.05,57.67) | 0.89(0.81,0.96) |
| Palau | 40.82(32.69,50.98) | 94.28(43.47,160.17) | 226.69(181.52,283.11) | 66.19(22.72,122.56) | 222.94(180.38,270.27) | -6.41(-29.96,23.92) | -0.1(-3.09,2.97) |
| Palestine | 3809.82(3325.69,4366.03) | 71.92(36.83,120.57) | 76.86(67.1,88.09) | -28.20(-42.86,-7.88) | 207.16(180.07,236.15) | -28.54(-42.26,-9.25) | -2.22(-2.53,-1.9) |
| Panama | 2510.96(1949.69,3135.67) | 58.03(26.55,94.07) | 60.35(46.86,75.37) | -9.28(-27.35,11.41) | 58.22(45.13,72.81) | -49.37(-59.49,-37.55) | -1.90(-2.41,-1.39) |
| Papua New Guinea | 7083.8(5169.16,9722.31) | 222.51(143.76,333.11) | 71.8(52.39,98.54) | 33.61(0.98,79.43) | 171.98(126.71,231.30) | 25.71(-0.93,61.95) | 0.92(0.70,1.14) |
| Paraguay | 4752.75(3731.89,5991.16) | 114.04(64.8,172.95) | 68.58(53.85,86.45) | 24.94(-3.8,59.33) | 89.6(70.25,112.93) | -17.82(-36.67,4.81) | -0.64(-0.97,-0.32) |
| Peru | 16047.61(11890.65,20492.24) | 42.34(4.36,91.79) | 47.21(34.98,60.28) | -9.01(-33.29,22.59) | 48.68(35.92,62.26) | -53.69(-66.04,-38.02) | -2.88(-3.02,-2.73) |
| Philippines | 97412.14(79700.14,114687.34) | 377.09(248.47,480.86) | 86.86(71.07,102.27) | 169.25(96.66,227.81) | 148.14(123.37,171.56) | 42.69(13.99,69.24) | 3.60(3.33,3.86) |
| Poland | 97188.36(81864.53,112078.91) | -24.41(-34.62,-13.42) | 252.87(213,291.61) | -24.95(-35.09,-14.04) | 130.31(110.03,150.64) | -59.52(-65.01,-53.71) | -4.98(-5.36,-4.60) |
| Portugal | 12989.95(11276.61,14223.89) | -18.28(-25.86,-11.22) | 121.96(105.87,133.54) | -22.22(-29.44,-15.51) | 45.77(40.39,49.87) | -63.98(-66.56,-61.34) | -4.8(-5.24,-4.36) |
| Puerto Rico | 4851.23(3800.1,5933.96) | -9.66(-27.17,10.73) | 137.76(107.91,168.51) | -7.31(-25.27,13.62) | 59.58(46.61,73.44) | -61.91(-69.56,-52.78) | -3.43(-3.95,-2.91) |
| Qatar | 830.01(630.61,1067.6) | 203.32(126.08,308.55) | 28.98(22.01,37.27) | -52.87(-64.87,-36.51) | 252.99(205.75,305.43) | -37.68(-50.16,-20.84) | -4.00(-4.61,-3.39) |
| Republic of Korea | 28280.7(23997.74,32634.65) | -9.27(-21.22,7.94) | 52.96(44.94,61.12) | -24.66(-34.59,-10.38) | 35.02(29.52,40.61) | -76.27(-79.21,-72.04) | -6.21(-6.40,-6.01) |
| Republic of Moldova | 15366.59(13476.99,17212.32) | 10.35(-1.6,22.95) | 416.64(365.41,466.69) | 33.03(18.62,48.23) | 265.43(232.66,297.19) | -35.52(-42.51,-28.32) | -1.19(-1.96,-0.42) |
| Romania | 69806.44(59148.37,81377.32) | 3.02(-12.02,19.05) | 362.87(307.47,423.02) | 25.29(7.01,44.8) | 177.15(150.34,206.81) | -41.23(-49.9,-32.07) | -2.83(-3.26,-2.40) |
| Russian Federation | 562609.02(488817.2,632969.35) | 14.5(1.56,27.74) | 383.46(333.17,431.42) | 17.86(4.54,31.49) | 240.6(208.85,270.82) | -23.74(-32.08,-15.05) | -1.73(-2.20,-1.25) |
| Rwanda | 3769.83(2754.95,4987.35) | 45.72(-1.24,93.78) | 29.71(21.71,39.31) | -17.62(-44.17,9.55) | 84.93(61.74,111.53) | -24.72(-47.99,-2.71) | -2.64(-2.90,-2.37) |
| Saint Kitts and Nevis | 55.18(48.21,62.89) | -35.94(-44.63,-25.22) | 92.73(81.01,105.68) | -55.48(-61.53,-48.04) | 104.07(91.83,117.32) | -57.67(-62.66,-51.18) | -3.92(-7.02,-0.72) |
| Saint Lucia | 133.74(115.6,153.77) | 13.12(-1.84,29.23) | 76.59(66.20,88.06) | -10.99(-22.76,1.68) | 66.59(57.61,76.35) | -58.01(-63.3,-52.12) | -3.21(-5.55,-0.82) |
| Saint Vincent and the Grenadines | 159.46(141.54,178.26) | 26.06(11.93,41.67) | 140.94(125.1,157.55) | 22.63(8.88,37.81) | 132.27(118.25,147.2) | -31.25(-38.61,-22.97) | -1.98(-3.98,0.06) |
| Samoa | 285.97(238.22,346.96) | 69.95(34.18,118.27) | 135.30(112.71,164.16) | 31.58(3.88,68.99) | 215.98(181.67,258.97) | -0.46(-20.4,24.96) | 0.08(-1.00,1.18) |
| San Marino | 37.04(25.78,49.59) | 49.03(2.6,103.51) | 111.89(77.87,149.81) | 6.06(-26.98,44.83) | 46.19(31.95,63.41) | -40.5(-59.7,-17.33) | -1.31(-9.86,8.04) |
| Sao Tome and Principe | 124.77(101.19,144.28) | 103.95(65.97,150.5) | 60.75(49.27,70.25) | 20.69(-1.79,48.23) | 153.15(124.47,176.56) | 28.5(6.72,53.5) | 0.37(-1.66,2.44) |
| Saudi Arabia | 29689.06(24089.22,36175.78) | 152.51(89.47,235.81) | 83.09(67.42,101.24) | 13.39(-14.92,50.79) | 205.6(172.88,238.97) | -14.8(-33.08,9.61) | -0.09(-0.26,0.08) |
| Senegal | 7130.94(5690.12,8748.56) | 109.5(67.06,164.46) | 47.12(37.6,57.81) | 5.49(-15.88,33.17) | 117.63(95.84,141.94) | -10.71(-26.84,9.04) | -0.49(-0.74,-0.24) |
| Serbia | 28365.38(23576.22,33585.3) | 17.9(-3.12,41.7) | 324.29(269.54,383.97) | 26.67(4.08,52.24) | 204.39(171.27,238.34) | -19.61(-33.07,-4.73) | -2.84(-3.62,-2.06) |
| Seychelles | 107.67(95.69,120.23) | 28.74(13.83,44.17) | 105.41(93.68,117.71) | -7.96(-18.62,3.07) | 110.19(97.60,122.93) | -26.96(-34.94,-18.63) | -1.71(-3.63,0.24) |
| Sierra Leone | 3920.25(2946.95,5056.85) | 63.73(31,108.95) | 47.32(35.57,61.04) | -27.83(-42.25,-7.89) | 134.01(103.83,170.09) | -8.09(-25.25,14.69) | 0.25(-0.08,0.58) |
| Singapore | 3833.28(3362.51,4135.36) | 34.73(22.28,44.33) | 67.64(59.33,72.97) | -27.56(-34.25,-22.4) | 52.34(45.61,56.67) | -65.32(-68.21,-63.01) | -3.88(-4.46,-3.30) |
| Slovakia | 17777.39(14344.1,21446.44) | -11.4(-28.36,6.28) | 326.96(263.81,394.44) | -13.91(-30.39,3.26) | 198.91(160.25,239.67) | -44.52(-55.09,-33.61) | -3.52(-4.02,-3.02) |
| Slovenia | 2979.77(2325.98,3848.14) | -8.26(-33.29,22.21) | 143.65(112.14,185.52) | -12.82(-36.61,16.13) | 59.04(46.20,76.19) | -58.57(-70.01,-44.5) | -4.56(-5.85,-3.26) |
| Solomon Islands | 1242.69(992.65,1503.64) | 138.02(80.08,212.93) | 189.54(151.40,229.34) | 23.60(-6.49,62.49) | 430.35(358.68,501.14) | 6.49(-15.93,33.98) | 0.20(-0.24,0.64) |
| Somalia | 6390.03(4725.7,8607.92) | 178.8(95.37,285.2) | 31.41(23.23,42.31) | -2.04(-31.36,35.34) | 124.60(94.03,166.56) | 7.22(-21.7,43.17) | 0.58(0.37,0.79) |
| South Africa | 30470.12(27595.63,32797.59) | 102.09(84.95,121.68) | 54.81(49.64,59) | 33.89(22.54,46.87) | 81.39(73.45,87.67) | 1.19(-7.30,11.05) | -1.60(-2.00,-1.20) |
| South Sudan | 2491.36(1743.79,3378.4) | 44.78(2.97,100.83) | 26.84(18.78,36.39) | -8.61(-35,26.76) | 84.24(60.14,111.51) | -5.94(-31.08,25.59) | -0.04(-0.39,0.31) |
| Spain | 53632.02(46434.2,59831.89) | -6.97(-13.80,1.28) | 116.54(100.9,130.01) | -21.61(-27.36,-14.65) | 44.96(40.03,49.47) | -59.46(-61.6,-56.48) | -3.66(-4.00,-3.33) |
| Sri Lanka | 23968.74(17942.29,30756.83) | 54.89(13.52,99.43) | 109.67(82.10,140.73) | 22.05(-10.55,57.15) | 109.05(81.57,138.94) | -37.56(-53.72,-20.04) | -2.26(-2.51,-2.00) |
| Sudan | 43187.36(33612.67,54936.11) | 46.98(18.00,82.98) | 105.83(82.37,134.62) | -27.25(-41.59,-9.43) | 271.47(214.92,338.38) | -24.96(-38.27,-9.04) | -1.62(-1.69,-1.55) |
| Suriname | 607.55(509.47,709.66) | 37.64(15.34,61.88) | 105.5(88.47,123.23) | -7.60(-22.57,8.68) | 106.90(89.91,124.63) | -40.82(-50.3,-30.61) | -2.10(-2.97,-1.23) |
| Sweden | 18974.61(16561.33,21084.89) | -39.32(-43.24,-33.15) | 185.62(162.01,206.26) | -49.02(-52.31,-43.84) | 73.66(65.51,81.20) | -61.19(-63.26,-57.60) | -4.11(-4.66,-3.57) |
| Switzerland | 12371.28(10379.69,14076.47) | -18.37(-26.22,-9.46) | 140.98(118.28,160.41) | -36.14(-42.28,-29.16) | 55.72(47.74,62.59) | -59.29(-62.49,-55.27) | -4.84(-5.40,-4.28) |
| Syrian Arab Republic | 33541.55(26238.92,43170.04) | 86.11(39.94,152.57) | 231.46(181.07,297.9) | 65.61(24.53,124.74) | 359.72(288.25,449.75) | -7.22(-28.38,22.88) | -1.53(-1.67,-1.39) |
| Taiwan (Province of China) | 16672.30(13460,20667.44) | 66.45(35.28,106.04) | 70.58(56.99,87.5) | 43.75(16.83,77.94) | 41.16(33.20,51.29) | -51.24(-60.55,-39.64) | -1.84(-2.11,-1.56) |
| Tajikistan | 13512.92(11366.26,16248.31) | 109.92(73.13,156.95) | 142.35(119.74,171.17) | 18.88(-1.95,45.51) | 439.18(376.64,515.79) | 82.10(53.66,116.80) | 0.37(-0.06,0.80) |
| Thailand | 51595.47(39018.99,65636.28) | 100.53(49.1,159.18) | 73.59(55.65,93.62) | 62.67(20.96,110.25) | 52.62(39.87,66.89) | -43.07(-57.29,-26.52) | -2.31(-2.54,-2.07) |
| Timor-Leste | 1020.85(768.96,1272.76) | 351.56(238.08,483.57) | 76.48(57.61,95.35) | 164.87(98.31,242.31) | 156.94(121.02,192.15) | 45.18(12.01,80.45) | 1.48(0.77,2.19) |
| Togo | 3709.21(2959.49,4712.95) | 176.07(123.02,252.7) | 46.82(37.36,59.5) | 27.66(3.13,63.09) | 134.88(111.77,166.54) | -5.40(-21.55,17.51) | 0.04(-0.27,0.35) |
| Tokelau | 2.31(1.89,2.86) | -6.53(-28.29,21.52) | 163.64(133.79,202.59) | 11.78(-14.24,45.33) | 188.65(154.74,232.17) | -2.12(-24.76,26.63) | -0.04(-12.53,14.23) |
| Tonga | 100.33(81.45,121.24) | 56.70(23.46,99.39) | 98.02(79.58,118.46) | 48.18(16.75,88.56) | 130.16(106.02,156.84) | -1.00(-21.39,24.90) | 0.14(-1.88,2.20) |
| Trinidad and Tobago | 2143.29(1657.68,2722.98) | 26.66(-1.52,61.35) | 154.48(119.48,196.26) | 9.82(-14.61,39.9) | 120.07(93.49,152.14) | -47.32(-58.9,-32.98) | -2.87(-3.48,-2.25) |
| Tunisia | 21457.03(16189.46,27280.55) | 126.78(68.72,196.98) | 185.43(139.91,235.75) | 65.39(23.05,116.59) | 193.45(146.86,244.04) | -18.99(-38.51,4.94) | -1.33(-1.49,-1.16) |
| Turkey | 99046.38(80454.39,120867.09) | 33.49(6.05,67.24) | 121.74(98.89,148.56) | -1.93(-22.09,22.87) | 120.96(97.99,147.20) | -47.38(-58.12,-34.73) | -3.88(-4.26,-3.50) |
| Turkmenistan | 11305.29(9342.94,13757.03) | 80.98(49.45,118.87) | 222.41(183.8,270.64) | 31.92(8.94,59.54) | 357.47(298.53,428.96) | -11.35(-25.94,5.97) | -1.61(-1.86,-1.36) |
| Tuvalu | 22.67(18.13,29.18) | 53.14(17.12,106.83) | 192.15(153.64,247.34) | 21.31(-7.22,63.84) | 251.53(201.8,317.74) | 1.77(-21.87,36.01) | 0.21(-3.54,4.10) |
| Uganda | 9590.28(6633.17,12166.69) | 129.93(81.74,188.65) | 23.32(16.13,29.59) | -3.17(-23.46,21.56) | 87.29(59.51,109.67) | 4.04(-16.14,29.85) | -0.11(-0.29,0.07) |
| Ukraine | 325873.55(284911.37,371595.62) | 50.26(32.95,70.78) | 739.91(646.9,843.72) | 79.67(58.97,104.20) | 424.23(369.92,483.93) | 24.33(10.1,40.43) | 0.79(0.33,1.26) |
| United Arab Emirates | 4880.13(3503.69,6754.69) | 482.44(302.29,721.68) | 52.81(37.91,73.09) | 17.98(-18.51,66.45) | 175.40(134.75,223.39) | -41.05(-54.12,-24.8) | -1.44(-1.77,-1.10) |
| United Kingdom | 93397.77(84321.48,98289.67) | -49.02(-51.66,-46.26) | 138.94(125.44,146.22) | -56.42(-58.67,-54.05) | 66.80(60.86,70.03) | -66.26(-67.68,-64.58) | -4.07(-4.46,-3.69) |
| United Republic of Tanzania | 18652.17(13645.86,23771.57) | 156.14(95.02,235.22) | 32.88(24.05,41.90) | 16.93(-10.97,53.04) | 95.36(70.75,119.6) | 7.3(-16.1,39) | 0.08(-0.06,0.22) |
| United States of America | 557648.79(496860.41,594411.46) | -7.69(-11.14,-3.43) | 170.03(151.49,181.23) | -28.62(-31.29,-25.33) | 91.03(82.44,96.54) | -49.86(-51.39,-47.60) | -2.25(-2.43,-2.07) |
| United States Virgin Islands | 295.84(262.98,326.28) | 111.16(80.96,147.88) | 284.5(252.9,313.77) | 115.28(84.49,152.71) | 174.5(155.35,192.7) | -14.98(-26.25,-1.70) | -1.16(-3.48,1.23) |
| Uruguay | 4090.92(3664.43,4373.52) | -30.71(-34.96,-26.61) | 119.06(106.64,127.28) | -36.7(-40.58,-32.95) | 67.02(60.9,71.32) | -56.77(-58.98,-54.36) | -2.89(-3.46,-2.31) |
| Uzbekistan | 75500.65(65705.82,86056.66) | 135.22(103.22,170.45) | 224.19(195.11,255.53) | 46.31(26.41,68.23) | 707.51(638.23,780.68) | 119.01(96.32,143.33) | 1.99(1.74,2.24) |
| Vanuatu | 460.14(358.37,605.25) | 207.33(133.93,311.46) | 156.22(121.67,205.48) | 57.95(20.23,111.47) | 304.91(243.14,388.28) | 15.81(-9.13,50.66) | 0.40(-0.42,1.24) |
| Venezuela (Bolivarian Republic of) | 36194.45(28429.71,46066.61) | 140.62(90.59,203.76) | 128.95(101.29,164.12) | 61.42(27.86,103.77) | 130.01(102.56,164.86) | -25.46(-41.11,-6.4) | -1.31(-1.49,-1.12) |
| Viet Nam | 74640.06(61449.95,88926.25) | 102.59(54.85,157.7) | 77.45(63.76,92.27) | 42.83(9.17,81.67) | 95.63(79.48,112.13) | -7.99(-28.56,15.60) | -0.8(-0.89,-0.71) |
| Yemen | 32304.75(26019.79,42118.22) | 122.47(75.47,194.46) | 102.55(82.59,133.7) | -3.05(-23.53,28.33) | 294.76(243.73,374.52) | -16.65(-32.34,5.21) | -1.11(-1.21,-1.01) |
| Zambia | 4833.05(3823.83,6056.29) | 95.67(42.89,159.7) | 26.5(20.97,33.21) | -14.78(-37.77,13.11) | 90.1(72.33,111.29) | -13.94(-36.28,11.58) | -1.11(-1.31,-0.90) |
| Zimbabwe | 8779.02(7036.93,10755.83) | 114.65(69.05,169.31) | 58.48(46.88,71.65) | 47.83(16.43,85.47) | 162.28(131.51,197.63) | 22.33(-2.68,52.90) | 1.61(1.35,1.86) |

**Notes:** Death numbers, death percentage change (1990-2019), all-age mortality rate in 2019, all-age mortality percentage change (1990-2019), age-standardized mortality rate in 2019, age-standardized mortality percentage change (1990-2019), and net drift of mortality from the APC model for all 204 countries.
